# Plasma extracellular vesicles reveal early molecular differences in amyloid positive patients with early-onset mild cognitive impairment

**DOI:** 10.1186/s12951-023-01793-7

**Published:** 2023-02-14

**Authors:** Amanda Cano, Ester Esteban-de-Antonio, Mireia Bernuz, Raquel Puerta, Pablo García-González, Itziar de Rojas, Claudia Olivé, Alba Pérez-Cordón, Laura Montrreal, Raúl Núñez-Llaves, Óscar Sotolongo-Grau, Emilio Alarcón-Martín, Sergi Valero, Montserrat Alegret, Elvira Martín, Pamela V. Martino-Adami, Miren Ettcheto, Antonio Camins, Assumpta Vivas, Marta Gomez-Chiari, Miguel Ángel Tejero, Adelina Orellana, Lluís Tárraga, Marta Marquié, Alfredo Ramírez, Mercè Martí, María Isabel Pividori, Mercè Boada, Agustín Ruíz

**Affiliations:** 1 Ace Alzheimer Center Barcelona – International University of Catalunya (UIC), C/Marquès de Sentmenat, 57, 08029 Barcelona, Spain; 2 Biomedical Research Networking Centre in Neurodegenerative Diseases (CIBERNED), Madrid, Spain; 3 Grup de Sensors I Biosensors, Departament de Química, Universitat Autònoma de Barcelona, 08193 Bellaterra, Spain; 4 Division of Neurogenetics and Molecular Psychiatry, Department of Psychiatry and Psychotherapy, Faculty of Medicine and University Hospital Cologne, University of Cologne, 50937 Cologne, Germany; 5 Department of Pharmacology, Toxicology and Therapeutic Chemistry, Faculty of Pharmacy and Food Sciences, University of Barcelona, 08028 Barcelona, Spain; 6 Institute of Neurosciences, University of Barcelona, Barcelona, Spain; 7 Departament de Diagnòstic Per La Imatge, Clínica Corachan, Barcelona, Spain; 8 Department of Neurodegenerative Diseases and Geriatric Psychiatry, University Hospital Bonn, Medical Faculty, 53127 Bonn, Germany; 9 German Center for Neurodegenerative Diseases (DZNE), 53127 Bonn, Germany; 10 Department of Psychiatry and Glenn, Biggs Institute for Alzheimer’s and Neurodegenerative Diseases, San Antonio, TX 78229 USA; 11 Cluster of Excellence Cellular Stress Responses in Aging-Associated Diseases (CECAD), University of Cologne, 50931 Cologne, Germany; 12 Biosensing and Bioanalysis Group, Institut de Biotecnologia I de Biomedicina (IBB-UAB), Mòdul B Parc de Recerca UAB, Campus Universitat Autònoma de Barcelona, 08193 Bellaterra, Spain

**Keywords:** Plasma exosomes, Extracellular vesicles, Cerebrospinal fluid, Proteomics, Alzheimer’s disease, Mild cognitive impairment

## Abstract

In the clinical course of Alzheimer’s disease (AD) development, the dementia phase is commonly preceded by a prodromal AD phase, which is mainly characterized by reaching the highest levels of Aβ and p-tau-mediated neuronal injury and a mild cognitive impairment (MCI) clinical status. Because of that, most AD cases are diagnosed when neuronal damage is already established and irreversible. Therefore, a differential diagnosis of MCI causes in these prodromal stages is one of the greatest challenges for clinicians. Blood biomarkers are emerging as desirable tools for pre-screening purposes, but the current results are still being analyzed and much more data is needed to be implemented in clinical practice. Because of that, plasma extracellular vesicles (pEVs) are gaining popularity as a new source of biomarkers for the early stages of AD development. To identify an exosome proteomics signature linked to prodromal AD, we performed a cross-sectional study in a cohort of early-onset MCI (EOMCI) patients in which 184 biomarkers were measured in pEVs, cerebrospinal fluid (CSF), and plasma samples using multiplex PEA technology of Olink^©^ proteomics. The obtained results showed that proteins measured in pEVs from EOMCI patients with established amyloidosis correlated with CSF p-tau^181^ levels, brain ventricle volume changes, brain hyperintensities, and MMSE scores. In addition, the correlations of pEVs proteins with different parameters distinguished between EOMCI Aβ( +) and Aβ(-) patients, whereas the CSF or plasma proteome did not. In conclusion, our findings suggest that pEVs may be able to provide information regarding the initial amyloidotic changes of AD. Circulating exosomes may acquire a pathological protein signature of AD before raw plasma, becoming potential biomarkers for identifying subjects at the earliest stages of AD development.

**Graphical Abstract:**

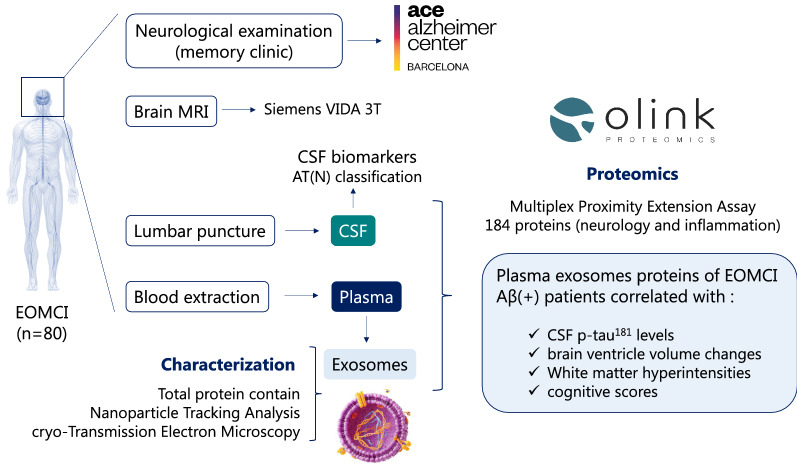

**Supplementary Information:**

The online version contains supplementary material available at 10.1186/s12951-023-01793-7.

## Highlights

- The pEVs proteome revealed early molecular differences between EOMCI Aβ( +) and Aβ(-) subjects.
- Olink^©^ neurology proteins from pEVs of EOMCI Aβ( +) patients exhibited a strong negative correlation with CSF p-tau^181^.
- The levels of pEVs and CSF Olink^©^ proteins correlated with CSF p-tau^181^ levels and age.
- Brain MRI characteristics of EOMCI Aβ( +) patients correlated with pEVs protein levels.
- pEVs biomarkers only linked early signs of inflammation to brain atrophy in EOMCI Aβ( +) individuals.
- The correlation of cognitive status and pEVs biomarkers differed between EOMCI Aβ( +) and Aβ(-) patients.

**Supplementary Information:**

The online version contains supplementary material available at 10.1186/s12951-023-01793-7.

## Introduction

The prevalence of dementia is rapidly growing due to social and demographic changes, mainly due to the increase in the population's longevity [1]. Consequently, the economic and social impact of the dementia epidemic threatens the sustainability of healthcare systems worldwide [2]. Alzheimer’s disease (AD) is the leading underlying cause of dementia in the elderly and is responsible for 60–80% of total dementia cases. AD is a neurodegenerative condition that progressively and irreversibly impairs cognitive functions, resulting in a complete loss of autonomy [3]. AD is the only condition among the 10 principal mortality causes worldwide that is still without a preventative treatment or cure. Currently, most AD cases are diagnosed after irreversible neuronal damage has occurred [4].

The pathophysiological course of AD begins with the formation of the first senile plaques, composed of extra-neuronal deposits of amyloid-β peptide (Aβ) and amyloid vascular deposits, and neurofibrillary tangles, composed of intra-neuronal deposits of hyperphosphorylated tau (p-tau). These pathological changes lead to synaptic dysfunction and, ultimately, neuronal loss, brain atrophy, and dementia It is accepted that AD pathogenesis has a very extensive preclinical stage in which the protein deposits occur silently and the manifestation of symptoms is not clinically appreciable. After this silent period, the cognitive performance of the individuals begins to decline [5]. Generally, memory and executive cognitive alterations appear first. This period corresponds to the mild cognitive impairment (MCI) phase of the disease. Notably, the MCI phase is mainly characterized by reaching the highest levels of Aβ and p-tau-mediated neuronal injury and pathological changes in the volumes of different brain regions. However, in this step, MCI subjects still retain the ability to perform daily life activities independently. Finally, when patients develop dementia, all these parameters reach their maximum levels and coexist simultaneously in the already irreversible stages of the disease [5]. At this point, neurological damage prevents the patient from having normal functionality and personal autonomy, and behavioral, cognitive, and memory alterations ultimately result in the patient’s death within approximately 10 years of diagnosis [2, 6].

Therefore, since many neurodegenerative diseases, including AD and non-neurodegenerative diseases, may present an MCI phase [7], a proper differential diagnosis in the prodromal stages of the disease is one of the greatest challenges in clinical practice. Apart from neurological and neuropsychological evaluations, AD diagnosis is based on neuroimaging, and cerebrospinal fluid (CSF) biomarkers [4]. However, the invasive nature and high cost of PET/CSF-biomarkers have promoted the growing scientific interest in peripheral biomarkers, including those derived from plasma and serum [8]. Although plasma biomarkers are showing very promising results, they have not yet been implemented in routine clinical practice and are still being studied [9]. Thus, given this scientific interest in plasma biomarkers, recent studies have focused on the potential utility of circulating plasma extracellular vesicles (pEVs) [10].

EVs are nanometric vesicles released by most cell types, including neurons, that contain proteins, lipids, metabolites, or RNA [11]. EVs play an important role in communication between neighboring cells and those of other tissues [12, 13]. Moreover, recent evidence has shown that EVs can also cross the blood–brain barrier (BBB) bidirectionally, thus enabling central and peripheral communication [14]. In pathological conditions, an overproduction of EVs has been described [11, 12]. This process is hypothesized to be a result of the increased need for cellular communication. It is also supposed to function as a signal for activating immune and non-immune processes, regenerating tissues, and recovering physiological homeostasis or as a spreading mechanism of disease hallmarks [12, 13]. However, the role of EVs in AD development is mostly unknown. A possible pathological signature of AD in circulating exosomes might be instrumental for early AD detection and could also provide further knowledge of the underlying molecular mechanisms. Many efforts are currently being made to elucidate the role of EVs in AD, both as pathology spreaders and as diagnostic tools through the early detection of their biomolecule profiles [15]. The leading research groups in the field of biomarkers have already reported that EVs can propagate Aβ pathology in cell cultures [16], are able to create clusters around the Aβ plaques [17], have reported that pEVs of AD patients show altered expression of proteins involved in AD pathogenesis [18–22], have identified abnormal levels of proteins in pEVs in MCI patients who converted to AD dementia [23].

To further investigate the role of circulating EVs in AD, we decided to initiate our own exosome research program. Here, we present a cross-sectional study on two groups of patients with early-onset MCI (EOMCI) (Aβ ±) from the BIOFACE cohort [24] in which we explored the potential of pEVs as an early diagnostic tool for AD by comparing the pEVs proteome profile to the paired CSF and plasma proteome of the same individuals.

## Materials and methods

### Standard protocol approvals, registrations and patient consents

All protocols of the BIOFACE study have been approved by the Clinical Research Ethics Commission of the Hospital Clinic (Barcelona, Spain) in accordance with the current Spanish regulations in the field of biomedical research and the Declaration of Helsinki. Likewise, in accordance with Spain’s Data Protection Law, all participants were informed about the study’s goals and procedures by a neurologist before signing an informed consent form. Patients’ privacy and data confidentiality were protected in accordance with applicable laws.

### Study participants and selection criteria

A total of n = 80 patients diagnosed at Ace Alzheimer Center Barcelona with EOMCI (under 65 years old) were included in the BIOFACE study [24, 25]. According to the International Working Group 2 criteria, subjects with altered biomarkers (a decrease in Aβ_1-42_ and an increase in total tau (t-tau) and phosphorylated tau proteins at threonine residue 181 (p-tau^181^) in CSF) were diagnosed with prodromal AD. The inclusion criteria were as follows: (i) age of onset between 50 and 65 years old; (ii) MMSE score ≥ 26; (iii) Clinical Dementia Rating (CDR) = 0.5; (iv) minimum elementary school level of education (≥ 6 years); (v) willingness to undergo a lumbar puncture; (vi) capacity to provide written informed consent; and (vii) fluency in Spanish. The exclusion criteria included: (i) contraindication for performing brain magnetic resonance imaging (MRI); (ii) active consumption of alcohol or drugs; (iii) known neurological diseases associated with cognitive impairment, such as Huntington’s disease, multiple sclerosis, or large vessel stroke; and (iv) limited capacity to provide informed consent.

### Brain MRI images

All BIOFACE patients underwent a brain MRI with a Siemens VIDA 3 T at Clínica Corachán’s Radiology Department, (Barcelona, Spain) at the baseline visit as described elsewhere [24]. MRI studies were examined by a group of experienced neuroradiologists and reported according to standard practice. The images were processed at Fundacio ACE ´ Neuroimaging Laboratory. All images were processed with Free surfer 6.0.1 (https://surfer.nmr.mgh.harvard.edu/).

### Plasma and CSF sample collection

All samples were collected at the baseline of the study. On the same day, plasma and CSF samples were collected from each patient. Blood samples were collected in polypropylene tubes with EDTA (BD Vacutainer). Plasma was separated by centrifugation (2000xg, 10 min, 4 °C), aliquoted, and stored at -80 °C until use. CSF was obtained by LP. An expert neurologist at Ace Alzheimer Center Barcelona performed LPs in accordance with established consensus recommendations [26]. The patient was fasted, placed in a sitting position, and anesthetized with 1% subcutaneous mepivacaine. In polypropylene tubes, 13 mL of CSF were collected (Sarstedt Ref 62.610.018). CSF was centrifuged for common AD biomarker determination (2000xg, 10 min, 4 °C), and the supernatant was aliquoted and stored at − 80 °C until use. The collection protocol followed the recommendations of the Alzheimer’s Biomarkers Standardization Initiative [27]. On the day of the analysis, an aliquot was thawed and used for the determination of Aβ_1-40_, Aβ_1-42_, t-tau, and p-tau^181^ proteins. Quantification was performed using a chemiluminescence enzyme immunoassay (CLEIA) with the Lumipulse G 600 II automatic platform (Fujirebio Inc.) [28].

### Isolation and characterization of pEVs

The gold standard ultra-centrifugation technique was used to isolate and purify pEVs from plasma samples [29]. In brief, 3.5 mL of plasma samples were centrifuged (10,000 g, 30 min, 4 °C) to remove cellular debris, and the supernatant was ultra-centrifuged twice (100,000 g, 60 min, 4 °C) to remove microvesicles and other debris, and then pellet the EVs. All centrifugations were done with a Sorvall Discovery M150 SE (Thermo Scientific) Ultracentrifuge using an S100AT6 rotor. The nanoparticle tracking analysis (NTA), measured using a NanoSight LM10-HS system with a tuned 405 nm laser (NanoSight Ltd., UK), was used to analyze the concentration and particle size of the pEVs. Cryogenic transmission electron microscopy (Cryo-TEM) was used to determine the morphology of exosomes. Images were collected by a Jeol JEM 2011 (JEOL USA Inc., USA) using an accelerating voltage of 200 kV. The total protein concentration of the obtained EVs was measured using the Pierce™ BCA Protein Assay Kit (Thermo Fisher).

### Proteomics

To prepare the pEVs samples for Olink^©^ proteomics and Pierce™ BCA total protein quantification, pEVs pellets were lysed with 40 µL of lysis buffer (50 mM TRIS pH 7.4, 150 mM NaCl, 1 mM EDTA pH 8, 1% Triton × 100, 0.01% sodium deoxycholate). Protein concentrations in CSF, plasma, and pEVs samples were quantified using the validated, highly sensitive, and specific ProSeek Multiplex immunoassay developed by Olink^©^ Proteomics (Uppsala, Sweden) as described elsewhere [30]. Biomarker measurements were conducted using multiplex Proximity Extension Assay (PEA) technology, following the manufacturer’s protocol [31]. In brief, 1 µl of samples were analyzed using two different commercially available ProSeek^®^ Multiplex panels, *Inflammation* (code 95302) and *Neurology* (code 95801), which allow the detection of 92 proteins from each panel simultaneously (Additional file 1: Figures S1 and S2). Antigens were incubated with pairs of antibodies containing DNA oligonucleotides bound to each of the 184 proteins to be measured [32, 33]. Oligonucleotides in close proximity produced a template for hybridization and extension. Pre-amplification was performed using a polymerase chain reaction method (PCR). Following digestion of residual primers, specific primers were digested on a quantitative real-time PCR chip (Dynamic Array IFC; Fluidigm Biomark) using a Biomark HD Instrument. Protein quantities were expressed as normalized protein expression (NPX) values on the log_2_ scale. Proteomic measurements of all the samples were carried out at the same time to avoid intra- and inter-assay variability.

### Statistical analysis

To perform a risk stratification study, subjects from each study group were classified into the [A/T/(N)] scheme by converting the CSF levels of Aβ_1-42_ (A), p-Tau^181^ (T), and t-Tau (N) into binary variables (abnormal, + ; normal, -) using in house proxy cut-off values [34]. Proxy cut-offs were obtained by plotting receiver operating characteristic (ROC) curves (CSF biomarker level as predictor and conversion as outcome) and calculating the Youden index, that is, the threshold value that provided the best tradeoff between sensitivity and specificity, using the *roc* and *coords* functions from the R package *pROC*. Since positive amyloidosis in CSF is a well-established indicator of an increased risk of phenoconversion to AD dementia [35], subjects were divided into Amyloid A( +) and A(-) groups for comparison. The NPX values for each protein in each sample were calculated as previously described [31] and were used as input for the data analysis pipeline.

Values below the LOD and proteins with over 25% missing values were excluded from the analysis. The quality control was performed on both the sample and protein levels. For sample QC, four internal controls were added to each sample to monitor the quality of assay performance as well as the quality of individual samples. The QC was performed in two steps: (1) only data with a standard deviation lower than that of the internal controls (0.2 NPX) were reported; and (2) only samples that deviated less than 0.3 NPX from the median value of the controls were considered to have passed the QC. Regarding protein QC, the detection limit was estimated from negative controls present on every plate, plus three standard deviations. The proteins that showed a missing data frequency of over 25% were also excluded (Additional file 1: Table S1).

Statistical analyses were conducted using GraphPad Prism 8.0 and R Studio. CSF levels of Aβ_1-42_, p-tau^181^, and t-tau were log_2_-transformed to normalize the data. Differences between the sexes were also analyzed. The Pearson correlation between age, sex, Qalb, MMSE, and CSF levels of log_2_-transformed Aβ_1-42_ and p-tau^181^ was calculated. After the correlation test, all values were Fisher *Z*-transformed to allow comparison of estimates:

1 $$Z=\mathrm{0,5}*(\mathrm{ln}\left(1+r\right)-\mathrm{ln}\left(1-r\right))$$

where r is the Pearson r correlation value.

## Results

### Characterization of pEVs and proteomics

**Fig. 1:**
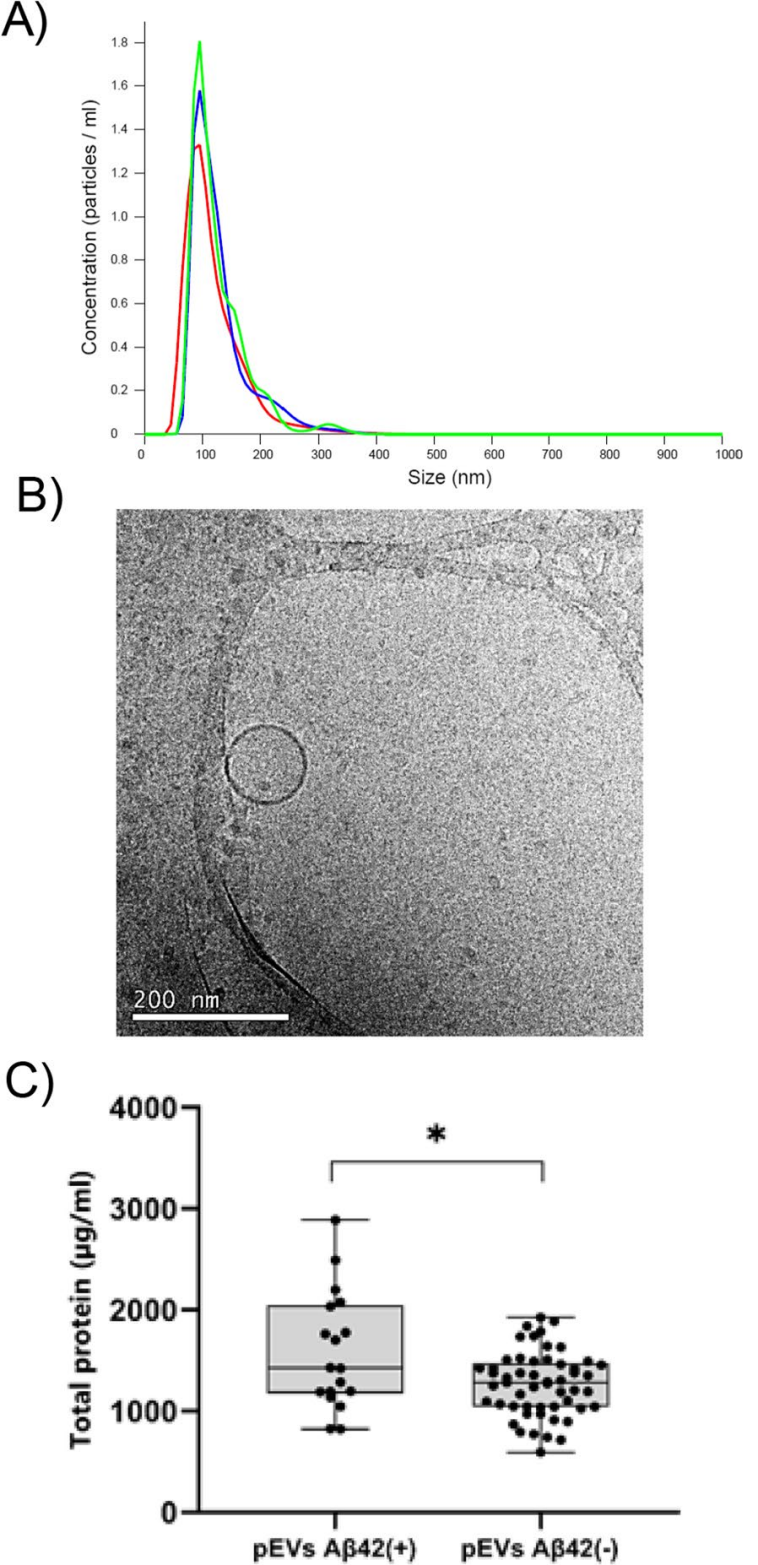
Characterization of pEVs. **A** Concentration of pEVs samples measured by NTA analysis. Concentration values are expressed as value^11. Measurements run by triplicate. **B** cryo-TEM image of isolated pEVs. Scale bar 200 nm. **C** Histogram shows the total protein concentration of the pEVs samples of EOMCI Aβ( +)/(-) patients. Statistical analysis was performed with an unpaired t test with Welch's correction. p = 0.0397; Difference between means ± SEM = 314.0 ± 143.0; CI (95%) = 611.8 to 16.21

The demographics and basic biochemistry of the BIOFACE cohort are display in Additional file 1: Table S2. NTA analysis revealed that pEVs samples had a homogenous particle population, with an average size of 98.3 ± 3.7 nm and a concentration of 1.018^11^ ± 3.782^9^ particles/ml (Fig. 1A). Cryo-TEM images showed isolated pEVs with a spherical shape, smooth surface particle and particle size in accordance with NTA analysis (Fig. 1B). Interestingly, the concentration of total protein showed statistically significant differences between pEVs samples of EOMCI Aβ( +) and EOMCI Aβ(-) patients (Fig. 1C). Olink^©^ technology detected 85 proteins in CSF, 91 in plasma and 77 in pEVs using the neurology panel, and 61 in CSF, 76 in plasma and 57 in pEVs using the inflammation panel (Additional file 1: Table S1).

### The pEVs proteome reveals early molecular differences between EOMCI Aβ( +) and Aβ(-) subjects

**Fig. 2:**
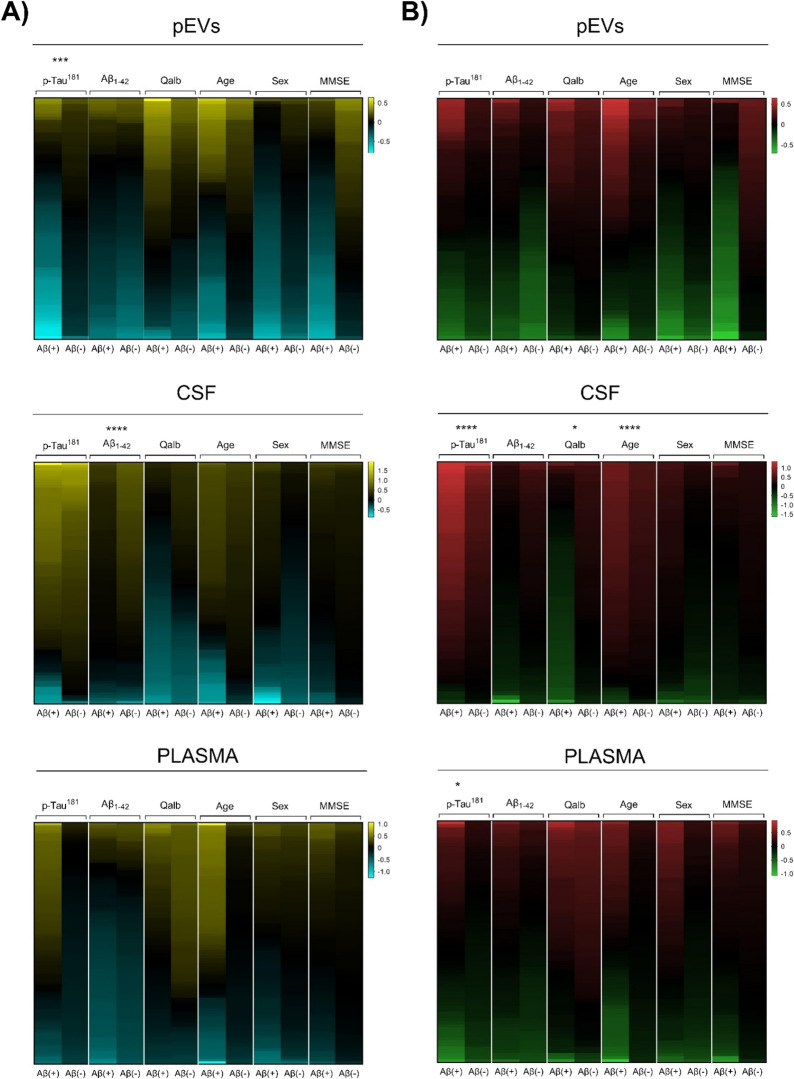
Heatmaps of the Pearson correlation’s of CSF p-Tau181, CSF Aβ1-42, Qalb, Age, Sex and MMSE levels *vs* **A** neurology biomarkers and **B** inflammation biomarkers in CSF, plasma and pEVs samples of Aβ( +) and Aβ(-) EOMCI patients. Statistical analysis was performed with Fisher’s exact test. Baptista-Pike method was used to compute CI’s. Correlations of interest were set at Pearson r > 0.5 and r < − 0.5. p < 0.05 (*). pEVs neurology—CSF pTau181: p = 0.0007; CI (95%) = 0.000 to 0.3355/CSF neurology—CSF Aβ1− 42: p =  < 0.0001; CI (95%) = 5.090 to Infinity/CSF inflammation—CSF pTau181: p =  < 0.0001; CI (95%) = 0.06201 to 0.3390/CSF inflammation—Qalb: p = 0.0275; CI (95%) = 0.000 to 0.6870/CSF inflammation—Age: p =  < 0.0001; CI (95%) = 0.000 to 0.1544/Plasma inflammation—CSF pTau181: p = 0.0135; CI (95%) = 0.000 to 0.6799

Pearson correlations were performed between Olink^©^ proteins measured in three compartments (CSF, plasma, and pEVs), two patient groups (EOMCI Aβ( +) and EOMCI Aβ(-)), and key AD endophenotypes (Additional file 1: Tables S3-S5). In the neurology panel, when comparing EOMCI Aβ( +) and EOMCI Aβ(-) groups, pEVs proteins exhibited statistically significant differences in their correlation with p-tau^181^, whereas CSF and plasma compartments did not (Fig. 2A). CSF only exhibited statistically significant differences in Aβ_1-42_ with neurology proteins. In the same way, when analyzing the Olink^©^ inflammation panel, pEVs were not able to show statistically significant differences in any of the key AD endophenotypes analyzed between Aβ( +) and Aβ(-) groups. However, some non-significant differences in the correlations were observed (Fig. 2B). CSF showed statistically significant differences in correlations with p-tau^181^, Qalb, and age, whereas plasma showed statistically significant differences only with p-tau^181^ and its inflammation proteins.

### The levels of pEVs and CSF Olink© proteins correlate with CSF p-tau^181^ levels and age

**Fig. 3:**
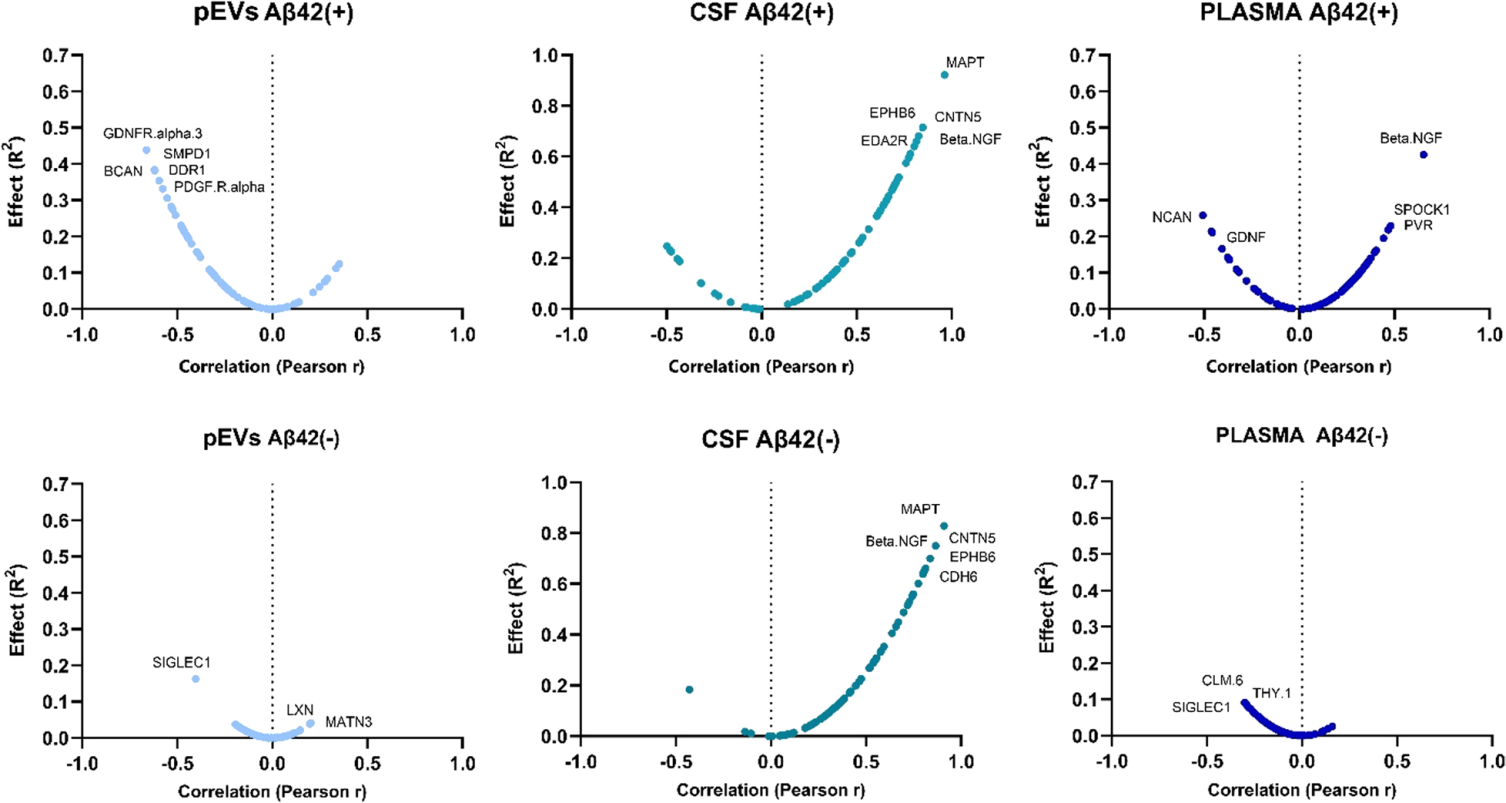
Volcano plots show the significance, expressed by the effect of correlation (R^2^) *vs* fold-change, expressed by Pearson r, of the correlation of neurology biomarkers *vs* CSF p-Tau181 levels

**Fig. 4:**
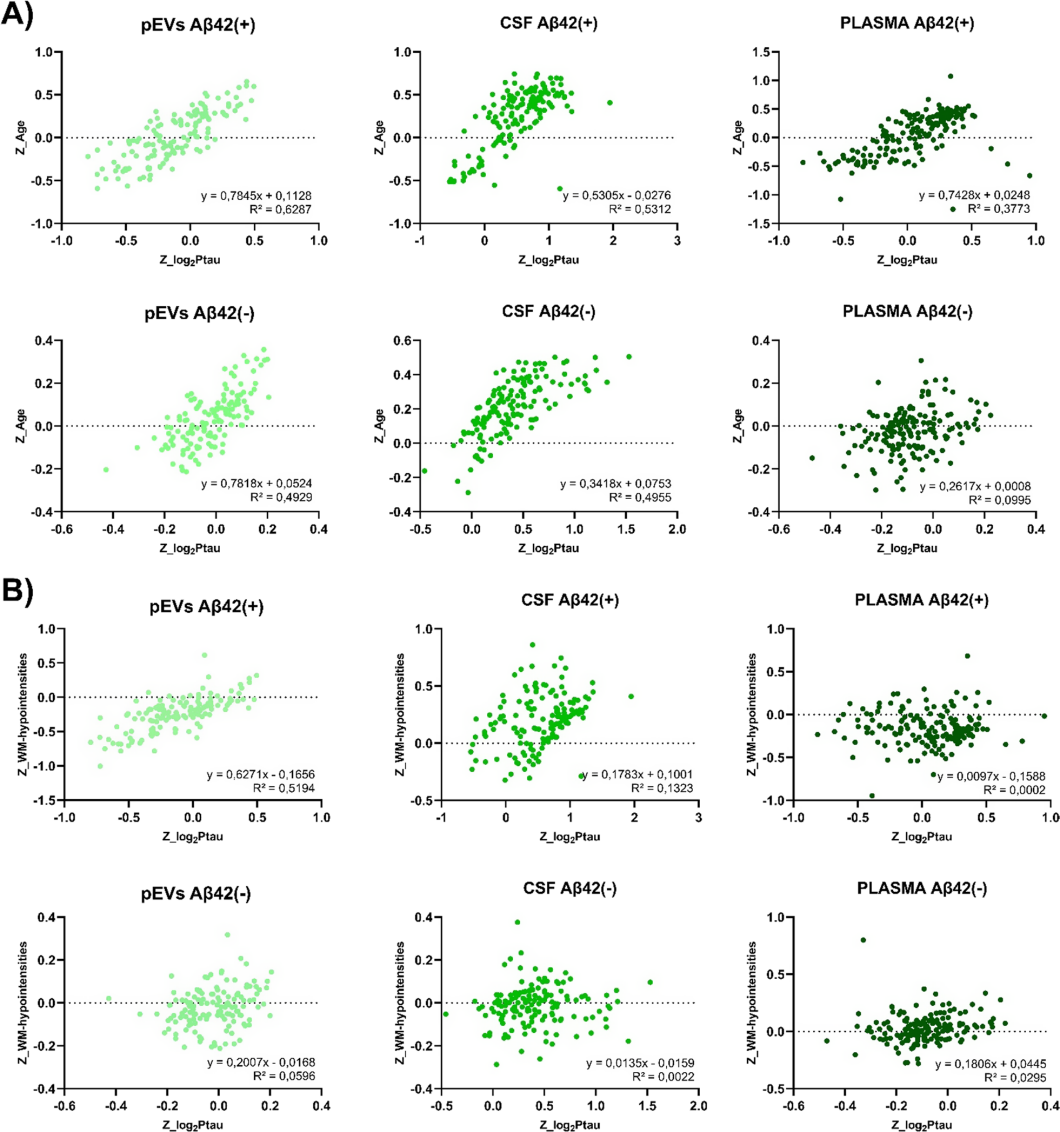
Graphs show the linear regression and equation parameters of the Pearson´s co-correlation of **A** the correlation of biomarkers *vs* CSF p-Tau181 and the correlation of biomarkers age; and **B** the correlation of biomarkers *vs* CSF p-Tau181 and the correlation of biomarkers white matter hypointensities in CSF, plasma and pEVs samples of EOMCI Aβ ( +)/(-) patients. Dots represent every single protein

When the magnitude of the effect and the degree of correlation of the biomarkers with p-tau^181^ were compared in the Olink^©^ neurology protein panels, the protein measured in pEVs of EOMCI Aβ( +) patients exhibited a strong negative correlation. In contrast, pEVs of EOMCI Aβ(-) patients did not exhibit any clear correlation with CSF p-tau^181^ (Additional file 1: Table S3). Intriguingly, the direct measurement of protein levels in CSF did not reveal any differences in correlations between EOMCI Aβ( +) and EOMCI Aβ(-) patients, with the same proteins exhibiting greater effects in both patient groups (Additional file 1: Table S4). Plasma exhibited no significant correlation, failing to distinguish between both EOMCI Aβ( +) and EOMCI Aβ(-) samples (Fig. 3). Of note, when comparing the global degree of correlation between Olink^©^ proteins with p-tau^181^ and age simultaneously, pEVs and CSF protein levels exhibited strong co-correlations (*R*^*2*^ > 0.5), whereas plasma could not exhibit this characteristic. Interestingly, pEVs of EOMCI Aβ( +) patients showed the highest concordance between protein level correlations with p-tau^181^ and age (*R*^*2*^ = 0.6287) (Fig. 4A). Moreover, when comparing the co-correlation of biomarkers to p-tau^181^ and white matter hypointensities (WMH), pEVs of EOMCI Aβ( +) patients also exhibited the strongest degree of co-correlation (*R*^*2*^ = 0.5194), whereas neither CSF nor plasma could show it (Fig. 4B).

### Brain MRI characteristics of EOMCI Aβ( +) patients correlate with pEVs protein levels

**Fig. 5:**
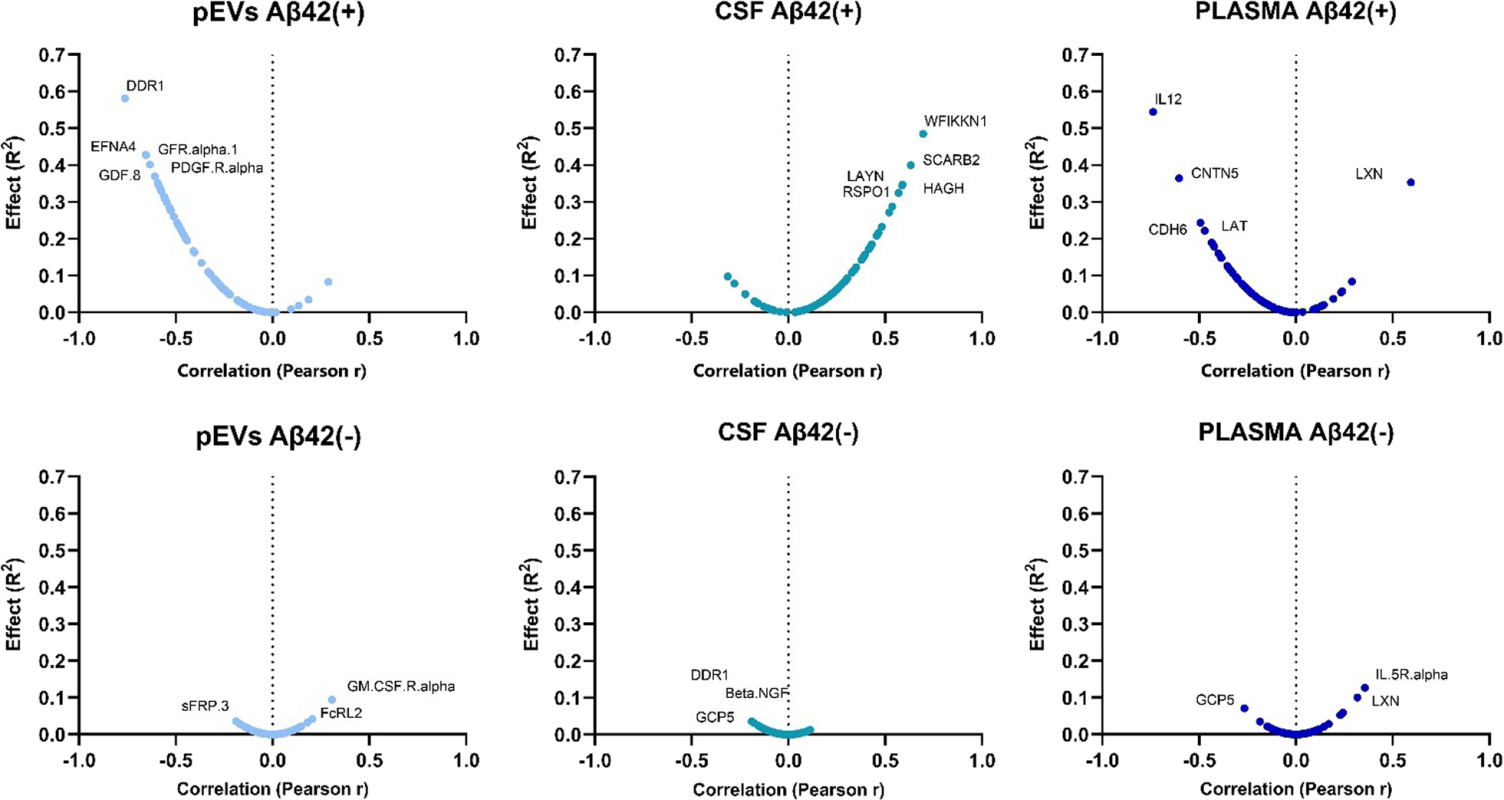
Volcano plots show the significance, expressed by the effect of correlation (R^2^) *vs* fold-change, expressed by Pearson r, of the correlation of neurology biomarkers *vs* white matter hypointensities

When comparing EOMCI Aβ( +) and EOMCI Aβ(-) patients’ MRI, differences in the volumes of several brain regions were not discernible. However, multiple neurology proteins demonstrated a clearly polarized correlation with WMH in CSF and pEVs (positive and negative, respectively) in EOMCI Aβ( +) patients, whereas EOMCI Aβ(-) patients exhibited no correlation (Fig. 5, Table 1). Plasma exhibited a similar but less pronounced pattern as pEVs. Moreover, both CSF and plasma did not show statistical differences in the correlation of their protein signatures and brain region volumes between EOMCI Aβ( +) and EOMCI Aβ(-) patients. Instead, several biomarkers from both the neurology and inflammation panels showed a significant correlation with the volume of certain brain regions in the pEVs samples of EOMCI Aβ( +) patients. Moreover, some proteins such as TRAIL, NTRK2, or PDGFR alpha exhibited correlations with different brain areas, whereas pEVs from EOMCI Aβ(-) patients did not (Table 1).

**Table 1:** Correlation between brain volumes and biomarkers that overcame FDR-correction from the BIOFACE cohort. p<0.001 (***), p<0.01 (**)

Brain region	Biomarker	Panel	Statistics	CSF_ Aβ42( +)	PLASMA_ Aβ42( +)	pEVs _ Aβ42( +)	CSF_ Aβ42(−)	PLASMA_ Aβ42(−)	pEVs _ Aβ42(−)
WM-hypo	DDR1	Neurology	R^2^	0.013	0.091	0.581	0.036	0.021	0.007
			Pearson r	0.116	− 0.302	− 0.7625	− 0.190898	− 0.144	− 0.086
			P value	0.648	0.239	0.000	0.143	0.278	0.540
			P summary	ns	ns	***	ns	ns	ns
Left Lat Ventr	PRTG	Neurology	R^2^	0.101	0.001	0.564	0.005	0.008	0.006
			Pearson r	− 0.317	0.026	0.751	− 0.068	0.087	− 0.079
			P value	0.200	0.921	0.001	0.600	0.514	0.562
			P summary	ns	ns	***	ns	ns	ns
	CPA2	Neurology	R^2^	0.015	0.001	0.532	0.004	0.005	0.048
			Pearson r	− 0.122	0.030	0.730	− 0.067	− 0.068	− 0.219
			P value	0.629	0.909	0.001	0.610	0.609	0.098
			P summary	ns	ns	***	ns	ns	ns
	PDGF-R-alpha	Neurology	R^2^	0.003	0.005	0.466	0.041	0.000	0.032
			Pearson r	− 0.050	− 0.073	0.682	− 0.203	0.015	− 0.179
			P value	0.844	0.781	0.003	0.115	0.910	0.187
			P summary	ns	ns	**	ns	ns	ns
	TRAIL	Inflammatory	R^2^	0.133	0.026	0.466	0.029	0.001	0.002
			Pearson r	− 0.364	− 0.161	0.682	− 0.169	0.026	0.041
			P value	0.137	0.524	0.002	0.193	0.841	0.758
			P summary	ns	ns	**	ns	ns	ns
	CX3CL1	Inflammatory	R^2^	0.060	0.105	0.483	0.001	0.014	0.000
			Pearson r	− 0.245	− 0.324	0.695	− 0.035	0.120	− 0.004
			P value	0.328	0.190	0.002	0.791	0.362	0.974
			P summary	ns	ns	**	ns	ns	ns
Right Lat Ventr	TRAIL	Inflammatory	R^2^	0.166	0.023	0.485	0.018	0.000	0.000
			Pearson r	− 0.407	− 0.153	0.696	− 0.133	0.018	0.011
			P value	0.094	0.545	0.001	0.307	0.892	0.931
			P summary	ns	ns	**	ns	ns	ns
Left Inf Lat Ventr	NTRK2	Neurology	R^2^	0.137	0.006	0.649	0.003	0.018	0.000
			Pearson r	− 0.370	0.079	0.787	− 0.051	− 0.132	0.003
			P value	0.131	0.764	0.000	0.698	0.318	0.984
			P summary	ns	ns	***	ns	ns	ns
4th-Ventricle	PDGF-R-alpha	Neurology	R^2^	0.070	0.060	0.596	0.002	0.000	0.007
			Pearson r	− 0.265	− 0.246	0.772	0.047	− 0.010	0.081
			P value	0.288	0.342	0.000	0.716	0.939	0.551
			P summary	ns	ns	***	ns	ns	ns
	MSR1	Neurology	R^2^	0.002	0.110	0.560	0.005	0.019	0.011
			Pearson r	0.044	0.332	0.749	0.070	0.137	0.105
			P value	0.862	0.193	0.000	0.593	0.301	0.433
			P summary	ns	ns	***	ns	ns	ns
	GZMA	Neurology	R^2^	0.011	0.023	0.508	0.007	0.038	0.033
			Pearson r	0.106	0.152	0.713	0.084	0.196	0.180
			P value	0.675	0.561	0.001	0.520	0.137	0.175
			P summary	ns	ns	***	ns	ns	ns
	SPOCK1	Neurology	R^2^	0.073	0.156	0.584	0.026	0.038	0.023
			Pearson r	− 0.270	0.395	0.764	− 0.161	− 0.195	− 0.153
			P value	0.279	0.116	0.001	0.215	0.139	0.284
			P summary	ns	ns	***	ns	ns	ns
	NTRK2	Neurology	R^2^	0.131	0.055	0.572	0.007	0.044	0.004
			Pearson r	− 0.362	0.234	0.756	− 0.086	− 0.210	− 0.060
			P value	0.140	0.365	0.001	0.509	0.110	0.674
			P summary	ns	ns	**	ns	ns	ns
	NTRK3	Neurology	R^2^	0.092	0.064	0.511	0.031	0.033	0.012
			Pearson r	− 0.303	0.252	0.715	− 0.176	− 0.182	− 0.111
			P value	0.222	0.329	0.001	0.174	0.167	0.418
			P summary	ns	ns	**	ns	ns	ns
	IL8	Inflammatory	R^2^	0.007	0.073	0.464	0.001	0.004	0.005
			Pearson r	− 0.086	0.271	0.681	0.028	0.065	0.069
			P value	0.735	0.278	0.002	0.830	0.622	0.605
			P summary	ns	ns	**	ns	ns	ns
	TRAIL	Inflammatory	R^2^	0.003	0.023	0.435	0.003	0.004	0.001
			Pearson r	− 0.057	0.150	0.660	− 0.055	− 0.066	− 0.029
			P value	0.822	0.552	0.003	0.676	0.616	0.827
			P summary	ns	ns	**	ns	ns	ns

### pEVs biomarkers only link early signs of inflammation to brain atrophy in EOMCI Aβ( +) individuals

**Fig. 6:**
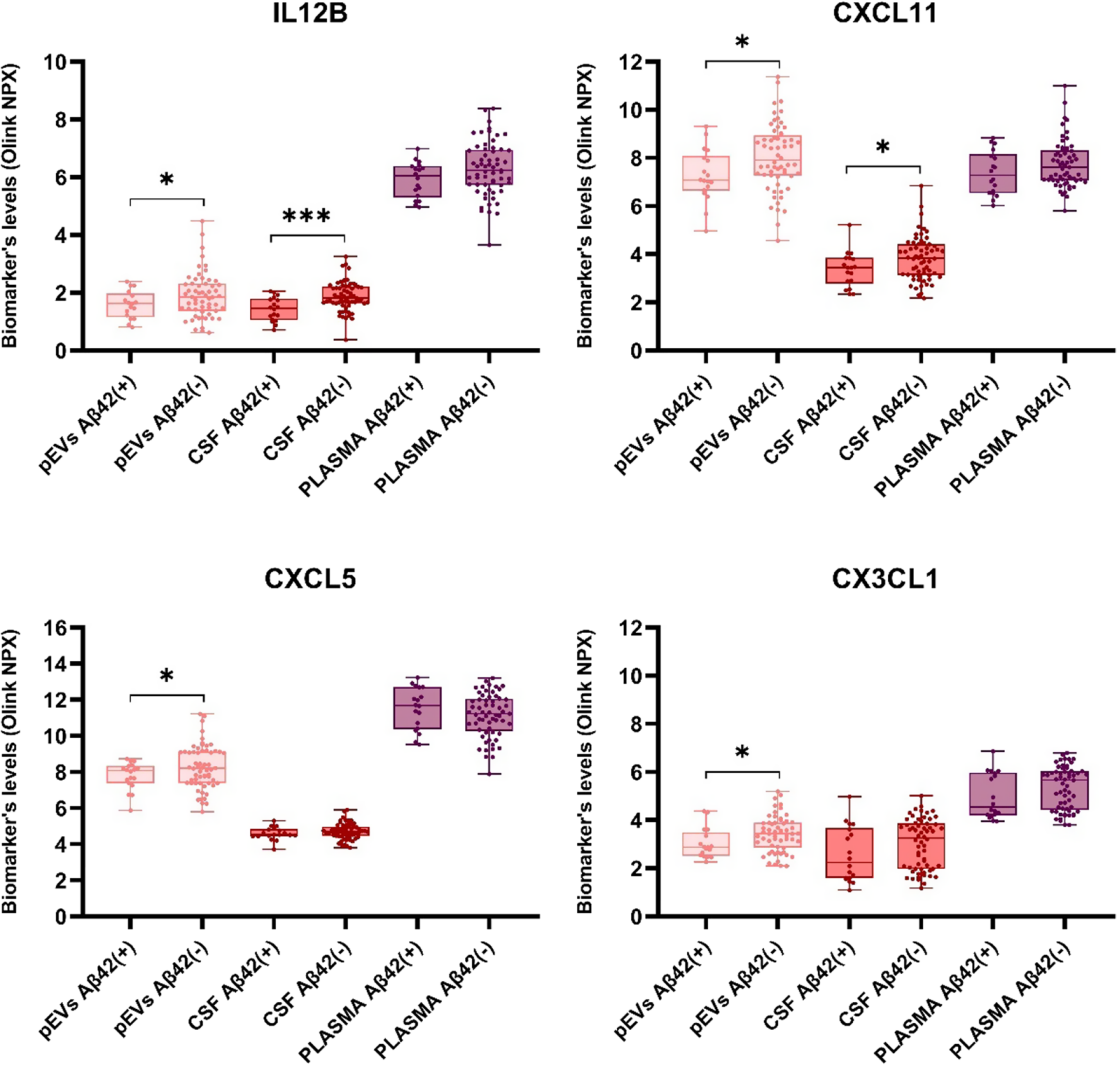
Histograms show the levels of several inflammatory biomarkers in CSF, plasma and pEVs samples of EOMCI Aβ ( +)/(-) patients. Statistical analysis was performed with an unpaired t test with Welch's correction. p < 0.05 (*). IL12B pEVs: p = 0.0465; CI (95%) = 0.0051 to 0.6309/IL12B CSF: p = 0.005; CI (95%) = 0.2104 to 0.6800/CXCL11 pEVs: p = 0.0201; CI (95%) = 0.1278 to 1.406/CXCL11 CSF: p = 0.0371; CI (95%) = 0.0287 to 0.8745/CXCL5 pEVs: p = 0.0486; CI (95%) = 0.0030 to 0,9610/CX3CL1 pEVs: p = 0.0369; CI (95%) = 0.0257 to 0.7659

**Fig. 7:**
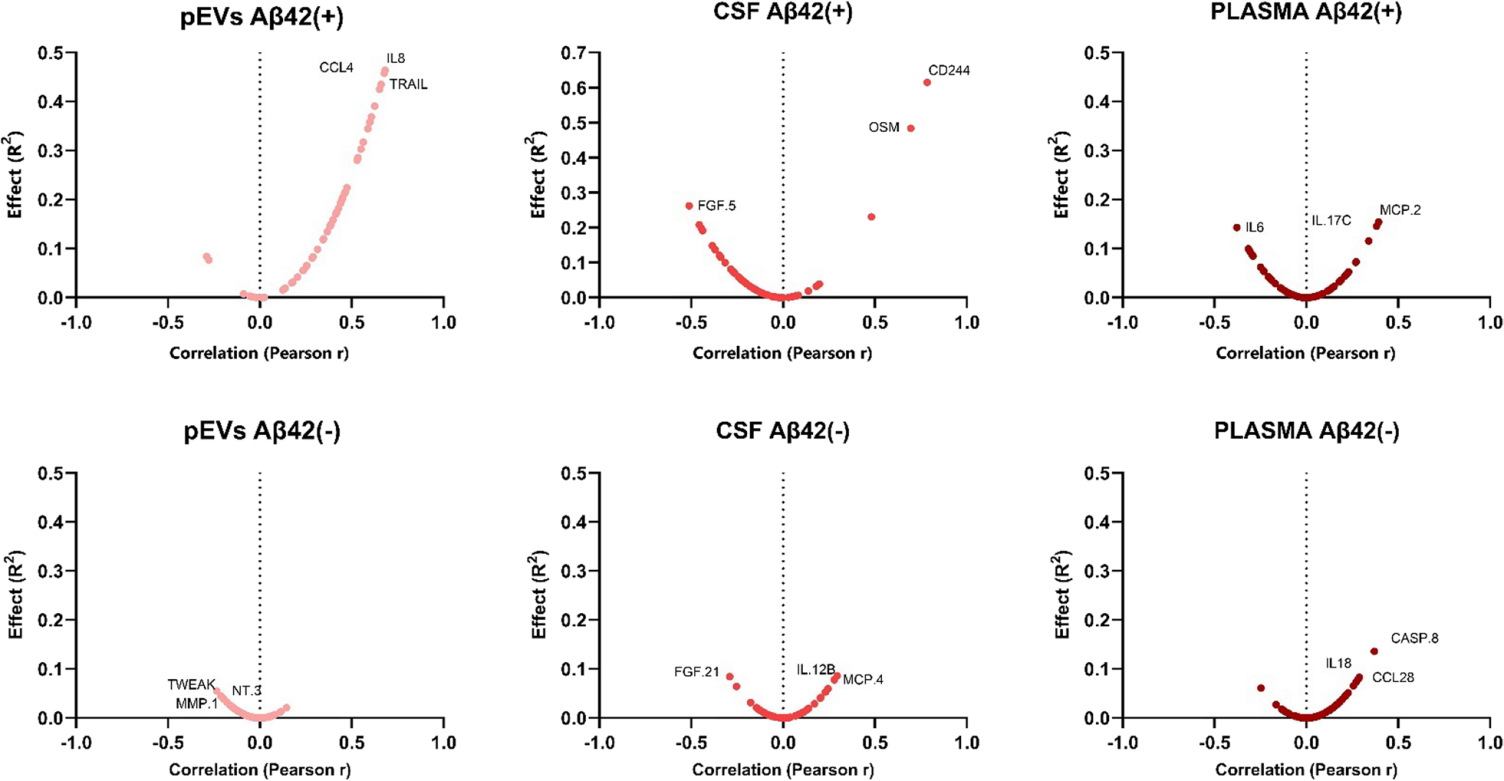
Volcano plots show the significance, expressed by the effect of correlation (R^2^) *vs* fold-change, expressed by Pearson r, of the correlation of inflammation biomarkers *vs* 4th ventricle volume

Several proteins of the inflammatory cascade showed statistically significant differences between EOMCI Aβ( +) and Aβ(-) patients in pEVs samples. Specifically, IL12B and CXCL11 were significant in CSF and pEVs compartments but not in plasma. Furthermore, CXCL5 and CX3CL1 only showed statistical differences in pEVs but not in CSF or plasma (Fig. 6). Ventricular enlargement is strongly correlated with a decline in cognitive performance, CSF, and pathologic markers of AD [36]. When comparing the correlation of inflammation protein with ventricle volumes, CSF and plasma did not show any significant correlation or difference between EOMCI Aβ( +) and Aβ(-) patients. In contrast, pEVs showed a clear difference, with a strong positive correlation of multiple proteins with brain ventricular volumes (Table 1) exclusively in the samples of EOMCI Aβ( +) patients (Fig. 7).

### The correlation of cognitive status and pEVs biomarkers differs between EOMCI Aβ( +) and Aβ(-) patients

**Fig. 8:**
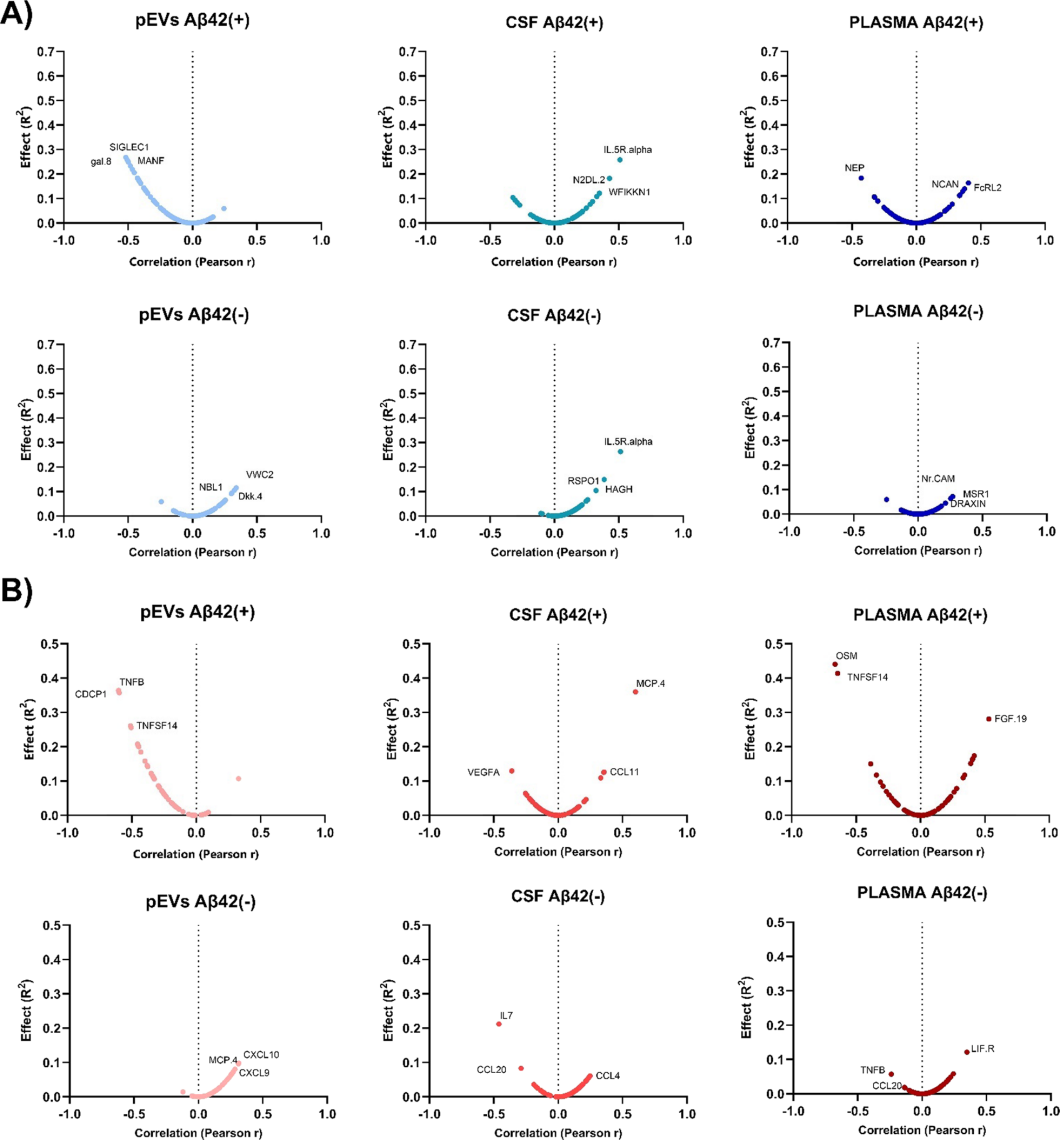
Volcano plots show the significance, expressed by the effect of correlation (R^2^) *vs* fold-change, expressed by Pearson r, of the correlation of **A** neurology biomarkers and **B** inflammation biomarkers *vs* MMSE

Neurology and inflammation biomarkers did not show a specific correlation with MMSE performance in both CSF and plasma samples. Moreover, these correlations were not able to differentiate between EOMCI Aβ( +) and Aβ(-) patients. In contrast, although the pEVs did not show a high degree of correlation, proteins from both the neurology and inflammation panels showed a negative correlation with MMSE in EOMCI Aβ( +) patients, which was absent in EOMCI Aβ(-) patients (Fig. 8).

## Discussion

Although it is well known that the first molecular alterations of AD pathogenesis can occur up to 15 years before the onset of clinical symptoms [37–39], a differential diagnosis in the early stage of AD development is still one of the greatest challenges nowadays. In this sense, non-invasive, cost-effective, population-wide pre-screening techniques based on the analysis of AD molecular hallmarks associated with early stages, including pre-amyloidotic stages, are necessary. Numerous efforts are currently being made to investigate the diagnostic potential of plasma biomarkers. Unfortunately, in these early stages, it is difficult to detect molecular alterations at the central level in plasma using current analysis methods. In addition, conventional plasma biomarkers currently available might not fully reflect AD status and are not very specific to AD pathophysiology [40–42]. For these reasons, EVs have garnered much interest as potential biomarkers since they are essential communication tools between neighboring cells and the periphery. Their protein, lipid, or mRNA content is directly related to the cellular processes between the cells involved and their environment. However, EVs profiles and their relationship with early AD processes are understudied, which prompted the development and conceptualization of this work. Thus, we performed a cross-sectional study in 80 patients with EOMCI Aβ42( +) and EOMCI Aβ42(-), analyzing 184 protein biomarkers of neurology and inflammation origin in paired samples of CSF, plasma, and pEVs.

Regarding the characterization assays, the results showed that the isolation process of pEVs was successful, yielding EVs with the same size, concentration, and shape as those described in previous studies [43–47]. Total protein content was higher in pEVs of EOMCI Aβ( +) patients than in Aβ(-) patients. MCI patients with Aβ( +) status are more likely to develop dementia than MCI Aβ(-) patients [48]. These results are in agreement with those described by Goetzl et al*.*, who found that the levels of numerous classical and alternative pathway complement effector proteins in astrocyte-derived plasma exosomes were significantly higher in patients with AD than in healthy controls [49]. Curiously, this is not limited to neurodegenerative diseases. Sharma et al*.* also found that total exosome protein levels, as measured by Pierce™ BCA, were higher in patients with melanoma than in healthy controls [46]. This could be related to the increase in cellular communication in pathological conditions. Overall, our findings support previous research that found increased EVs biogenesis in pathological conditions [11, 12].

In relation to AD endophenotypes and covariates, our results showed a correlation between the p-tau^181^ levels in CSF and pEVs biomarkers. When analyzing the shape of the correlations between the protein levels of both panels and the main AD parameters, the pEVs showed clear differences between EOMCI Aβ( +) and Aβ(-) patients, highlighting p-tau^181^. When comparing the correlation between neurology biomarkers and p-tau^181^, plasma showed no clear correlation or difference between both subgroups, and CSF showed a clear positive correlation in both cases; pEVs were able to show a clear difference between the pattern of correlation between EOMCI Aβ( +) and Aβ(-) patients. Intriguingly, EOMCI subjects with positive amyloidosis showed a fraction of proteins displaying negative correlations between neurology biomarkers and CSF levels of p-tau^181^ in pEVs, suggesting an AD-specific signature of neurodegeneration in the plasmatic EVs compartment. This negative correlation suggests the hypothesis that the emerging amyloidosis state contributes in some way to a blackout in the neuron-derived EVs proteome. Importantly, these results are somehow in agreement with those found by Goetzl et al*.,* who identified four proteins of neuronal origin (GluA4-containing glutamate receptor, neuronal pentraxin 2, neurogranin, and neurexin 2α) that were significantly decreased in the neuronal-derived pEVs of AD patients compared to those of healthy controls [50]. Researchers at the Boston University School of Medicine showed that AD brain-derived exosomes could also spread tau pathology in healthy mouse interneurons [51]. Their study proposed a novel mechanism for the spread of tau in hippocampal GABAergic interneurons through brain-derived extracellular vesicles and their subsequent neuronal dysfunction. Our results showed no significant differences in microtubule-associated protein tau (MAPT) levels between EOMCI Aβ( +) and EOMCI Aβ(-) patients. In plasma samples, MAPT protein was undetectable by the Olink^©^ panel. Our results showed that Olink^©^ technology was likely insufficiently sensitive for this purpose at these early stages. These findings could be related to the previously described disseminative role of EVs and could be one of the distinctive hallmarks of the beginning of AD molecular alterations at these early stages.

Together with other pathophysiological factors, tau phosphorylation, which is primarily induced by the formation and accumulation of Aβ oligomers, initiates the neurodegenerative process. These alterations also lead to the deregulation of other proteins and cell types in the brain [52]. In this context of amyloidosis and tau phosphorylation, our results showed that several neurology biomarkers in pEVs samples were highly negatively correlated with CSF p-tau^181^ only in EOMCI Aβ( +) patients. Most of them are involved in cell growth, synaptic plasticity, and neuron-extracellular matrix communication.

Specifically, among the proteins related to growth factors, we found that GDNFR alpha 3 (*r* = − 0.662/*R*^*2*^ = 0.4382) and PDGFR alpha (*r* = − 0.5947/*R*^*2*^ = 0.3319) decreased in pEVs of EOMCI Aβ( +) patients with the highest levels of CSF p-tau^181^. In both cases, previous studies have pointed out that both protein levels are significantly altered in MCI and AD patients [53], even in early-stage AD patients, suggesting an adaptive process of the impaired brain [54]. Our results also highlight the importance of both pathways. Interestingly, pEVs PDGFR alpha not only showed a strong negative correlation with p-tau^181^ levels in EOMCI Aβ ( +) patients, but also with WMH (r = − 0.6340/R^2^ = 0.4019), the main hallmark of brain vascular impairment. Likewise, pEVs PDGFR alpha also appeared to be strongly correlated with different ventricle volumes only in EOMCI Aβ ( +) patients (see Table 1).

Regarding the proteins involved in the communication of neurons with the extracellular matrix, DDR1 (*r* = − 06.179/*R*^*2*^ = 0.3818) and BCAN (*r* = − 0.6196/*R*^*2*^ = 0.3840) were the most highly correlated in the Aβ( +) group. Previous studies have identified co-correlated peptide clusters associated with varying levels of p-tau. Many of these are involved in plasticity and extracellular matrix remodeling, including DDR1, suggesting that they could be involved in the tissue reaction around Aβ plaques [55]. This could be related to the reduced levels found in pEVs of EOMCI Aβ( +) patients but not in Aβ(-) patients. In addition, our results showed a strong negative correlation between DDR1 levels and WMH (*r* = − 0.7625/*R*^*2*^ = 0.5813) only in EOMCI Aβ( +) patients. Related to Brevican (BCAN), Jonesco et al*.* demonstrated in a cross-sectional study that this protein exhibits differential serological levels in AD, other types of dementia, and non-dementia patients [56]. Similarly, Minta et al*.* were able to discriminate between AD and vascular dementia patients by measuring the BCAN concentration in CSF [57]. Since EVs s are a means of communication for neuronal cells, the impairment in the synaptic transmission induced by Aβ and p-tau could be related to the reduced levels of BCAN found in pEVs of patients with already established amyloidosis.

SPOCK1, also called Testican-1, has also been related to synaptic function and cell communication and is mainly expressed in neurons and oligodendrocytes, but its function is still unknown. Our results show decreased levels of SPOCK1 in pEVs of EOMCI Aβ ( +) patients with increased CSF p-Tau^181^ (r = − 0.5532/R^2^ = 0.3060). Another altered protein in pEVs of EOMCI Aβ( +) patients was sphingomyelin phosphodiesterase 1 (SMPD1), a lysosomal acid sphingomyelinase that converts sphingomyelin to ceramide. Our results demonstrated a highly negative correlation of SMPD1 with p-tau^181^ in the pEVs compartment of EOMCI Aβ( +) patients (*r* = − 0.6178/*R*^*2*^ = 0.3817). Finally, contactin-5 protein (CNTN5) also appeared to have a strong inverse correlation with CSF p-tau^181^ levels (*r* = − 0.5761/*R*^*2*^ = 0.3319) in CSF and pEVs but not in plasma. Tedeschi Dauar et al*.* evaluated the association of CNTN5 genotype and protein levels with pathological hallmarks and clinical manifestations of AD. Their results highlighted that the rs146168 variant of CNTN5 plays a role in the risk of developing AD, and that CNTN5 CSF levels are strongly correlated with AD pathology, especially in the pre-symptomatic phase of the disease [58]. Increased tau phosphorylation and resulting axonal damage could be behind this reduction in CNTN5 levels observed in pEVs.

The relationship between EVs biomarkers and vascular impairment was another fascinating finding. Growing scientific evidence suggests that the vascular health hypothesis plays a significant role in the development of AD [59]. Our results showed that all fluids correlated with WMH and neurology biomarkers in EOMCI Aβ( +) patients, being negative in the case of pEVs and plasma and positive in CSF, whereas EOMCI Aβ(-) patients did not exhibit any significant correlation in any fluid. The inverse correlation between the central fluid and pEVs is remarkable. Furthermore, when comparing the correlations of WMH and p-tau^181^ to neurology biomarkers, only pEVs from EOMCI Aβ( +) patients exhibited a strong co-correlation. All of these findings support the hypothesis that pEVs might contain information about a specific signature of AD neurodegeneration, which is also related to early vascular alterations in the initial stages of AD [60].

The inflammation process is one of the typical hallmarks of these early stages of AD pathology. Intriguingly, pEVs also showed some differences that CSF and plasma did not. Firstly, pEVs of EOMCI Aβ( +) and Aβ(-) subjects exhibited different levels of chemokines and interleukins previously implicated in AD-like pathology and cognitive decline, whereas plasma and some CSF did not. These proteins were CXCL5, CX3CL1, IL12B and CXCL11. Curiously, using the same technology and neurology/inflammation panels of Olink^©^ proteomics, Nielsen et al*.* recently evaluated the protein cargo of plasma-derived EVs in patients with AD, MCI, and healthy controls [61]. Their results demonstrated that CCL11 showed diagnostic capabilities between healthy controls and AD in EV samples. Regarding IL12B, many authors have highlighted the association between abnormal IL12 levels and AD. Vom Berg et al*.* reported a decade ago that the inhibition of IL-12/IL-23 signaling reduced AD-like pathology and cognitive decline [62]. An outstanding study by Pedrini et al*.* revealed that IL-10 and IL-12/23p40 were jointly associated as predictors of amyloid-β load in AD patients [63]. Similarly, Johansson et al*.* showed that CSF IL-12/23 p40 concentration was decreased in AD and MCI patients compared to healthy controls [64]. Lin et al*.* found that polymorphisms in IL-12-associated genes (including the rs730691 variant in the IL12B gene) were associated with cognitive aging [65]. Our results were in agreement with those findings and could reflect the ability of EVs to demonstrate the well-described inflammation process that occurs in these early stages.

In relation to brain volumes, whereas CSF and plasma were unable to demonstrate a correlation between inflammatory biomarkers and ventricle volumes in EOMCI Aβ( +) and Aβ(-) patients, pEVs of EOMCI Aβ( +) patients exhibited a positive correlation, as evidenced by an enlargement of the 4th ventricle and an increase in the levels of inflammatory biomarkers. It is tempting to speculate that pEVs could provide information about brain atrophy processes even before structural MRI could detect the differences in volume measurements at this early stage. These results are in agreement with previous findings that highlighted ventricular enlargement as an objective and sensitive measure of neuropathological changes associated with MCI and AD progression [66, 67]. Likewise, the relationship between inflammation biomarkers and ventricle volumes has also been described. Thus, Walker et al*.* evaluated the relationship between systemic inflammation and neurodegeneration by measuring circulating inflammatory markers and different brain volumes and found that an increased inflammation composite score was associated with 1788 mm^3^ greater ventricular volume [68].

The MMSE is a well-known and used short screening tool for providing an overall measure of cognitive impairment in clinical settings. However, the MMSE cannot be used as a standalone test to identify MCI patients at risk of developing dementia [69]. The combination of a detailed clinical history, cognitive tests, and biomarker analysis provides a comprehensive diagnosis for patients in the MCI phase. In our study, the MMSE was unable to show statistically significant differences between EOMCI Aβ( +) and Aβ(-) patients. However, its correlations with both neurology and inflammation biomarkers in pEVs differed between patients with established amyloidosis and those who did not have the disease. This could be a combined indicator of the cognitive decline that precedes the advanced phases of the disease’s development, but its relationship is not entirely clear and requires further investigation.

However, our study has some limitations. The restricted sample volume and concentration of the pEVs samples significantly conditioned the development of proteomic assays. Due to the volume/concentration requirements of the proteomics platforms, the full sample was intended for the Olink^©^ proteomics screening assays. Likewise, the sample size was restricted to 80 individuals due to the complexity of recruiting patients for the study. Although the EVs isolation process is one of the gold standards (ultracentrifugation), this technique would not be feasible for large-scale studies due to the high volume of sample required (3.5 ml). Due to the exploratory nature of this study, which aimed to evaluate a general AD signature in circulating pEVs, total EVs were selected. However, the use of specific cell-derived EVs would be of interest for future assays to investigate more specific molecular pathways related to the physiopathological processes of the disease development. However, despite being preliminary, our results connect Aβ( +) AD status with some key endophenotypes, suggesting that pEVs protein content could be related explicitly to early AD processes. Although further molecules must be analyzed in search of a specific “EVs AD signature,” we found intriguing evidence that EVs may reflect early molecular alterations of the neurodegenerative process. In this regard, additional research is required to validate our findings in independent series and identify direct relationships between specific pEVs proteins and AD endophenotypes, including disease progression.

## Conclusions

At the earliest stages of AD development, molecular alterations are nearly undetectable in complex matrices such as CSF or plasma. Our findings suggest that EVs may contain very specific information about the molecular processes occurring in their originating cells and microenvironment. These vesicles are capable of providing this molecular information, whereas CSF and plasma are not. In summary, our results suggest that pEVs have the potential to reveal the molecular events preceding clinical decline. At this early stage, our results may indicate that their biomarker profile is associated with the AD-specific neurodegeneration process governed by p-tau and Aβ, premature neuroinflammation processes, and brain atrophy. In addition, their peripheral accessibility and low invasiveness make pEVs a potential source of biomarkers for screening purposes. Thus, the current work sheds light on the search for new peripheral biomarkers for the early diagnosis of AD. However, additional research is required to comprehend the molecular pathways underlying these findings and validate the obtained results in an independent cohort.

## Supplementary Information

**Additional file 1: Figure S1.** Neurology panel of Olink Proteomics (code 95801). Distribution of analytical measuring range, defined by the lower and upper limits of quantification (LLOQ-ULOQ), and normal plasma levels where data is available (dark blue bars) for 92 analytes. **Figure S2.** Inflammation panel of Olink Proteomics (code 95302). Distribution of analytical measuring range, defined by the limits of quantification LLOQ-ULOQ, for 90 out of 92 analytes. **Table S1.** List of proteins excluded/included in the study after the quality control analysis. **Table S2.** Demographics and biochemistry of the BIOFACE cohort. **Table S3.** Pearson correlations and effect between biomarkers in pEVs and most common parameters of AD analysis. Data ordered by R2. Displayed proteins above R2 > 0.3. **Table S4.** Pearson correlations and effect between biomarkers in CSF and most common parameters of AD analysis. Data ordered by R2. Displayed proteins above R2 > 0.3. **Table S5.** Pearson correlations and effect between biomarkers in plasma and most common parameters of AD analysis. Data ordered by R2. Displayed proteins above R2 > 0.3.

## Data Availability

The data that support the findings of this study are available from the corresponding authors upon reasonable request.

## Abbreviations

Aβ: Amyloid β
AD: Alzheimer’s disease
BBB: Blood-brain barrier
CLEIA: Chemiluminescense enzyme immunoassay
CSF: Cerebrospinal fluid
DALYs: Disability-adjusted life years
EOMCI: Early-onset mild cognitive impairment
EVs: Extracellular vesicles
LP: Lumbar puncture
MCI: Mild cognitive impairment
MRI: Magnetic resonance imaging
NPX: Normalized protein expression
NTA: Nanoparticle Tracking Analysis
pEVs: Plasma extracellular vesicles
PCR: Polymerase chain reaction
p-tau: Hyperphosphorylated tau
t-Tau: Total Tau
WMH: White matter hypointensities

## References

[1] Nichols E, Szoeke CEI, Vollset SE, Abbasi N, Abd-Allah F, Abdela J (2019). Global, regional, and national burden of Alzheimer ’ s disease and other dementias, 1990–2016: a systematic analysis for the Global Burden of Disease Study 2016. Lancet Neurol.

[2] Alzheimer’s Association (2021). Alzheimer’s disease facts and figures. Alzheimers Dement.

[3] Jack CR, Bennett DA, Blennow K, Carrillo MC, Dunn B, Haeberlein S, Holtzman DM (2018). Toward a biological definition of Alzheimer’s disease. Alzheimers Dement.

[4] Cummings J, Isaacson R, Schmitt F, Velting D (2015). A practical algorithm for managing Alzheimer’ s disease: what, when, and why ?. Ann Clin Transl Neurol.

[5] Sperling RA, Aisen PS, Beckett LA, Bennett DA, Craft S, Fagan AM (2011). Toward defining the preclinical stages of Alzheimer’s disease: recommendations from the National Institute on Aging-Alzheimer’s Association workgroups on diagnostic guidelines for Alzheimer’s disease. Alzheimers Dement.

[6] Joling KJ, Janssen O, Francke AL, Verheij RA, Lissenberg-Witte BI, Pieter-Jelle V (2020). Time from diagnosis to institutionalization and death in people with dementia. Alzheimers Dement.

[7] Alzheimer’s Association. More than normal aging: understanding mild cognitive impairment. 2022. https://www.alz.org/media/Documents/alzheimers-facts-and-figures-special-report.pdf

[8] Zetterberg H (2019). Blood-based biomarkers for Alzheimer’ s disease — An update. J Neurosci Methods.

[9] Teunissen CE, Verberk IMW, Thijssen EH, Vermunt L, Hansson O, Zetterberg H (2021). Blood-based biomarkers for Alzheimer’s disease: towards clinical implementation. Lancet Neurol.

[10] Schöll M, Maass A, Mattsson N, Ashton NJ, Blennow K, Zetterberg H (2019). Biomarkers for tau pathology. Mol Cell Neurosci.

[11] de Toro J, Herschlik L, Mongini C, Waldner C (2015). Emerging roles of exosomes in normal and pathological conditions: new insights for diagnosis and therapeutic applications. Front Immunol.

[12] Kalluri R, LeBleu VS (2020). The biology, function, and biomedical applications of exosomes. Science.

[13] Cano A, Ettcheto M, Bernuz M, Puerta R, Esteban de Antonio E, Souto EB (2023). Extracellular vesicles, the emerging mirrors of brain physiopathology. Int J Biol Sci.

[14] Wood MJ, O’Loughlin AJ, Lakhal S (2011). Exosomes and the blood–brain barrier: implications for neurological diseases. Ther Deliv.

[15] Zhang T, Ma S, Lv J, Wang X (2021). The emerging role of exosomes in Alzheimer’ s disease. Ageing Res Rev.

[16] Sardar M, Anna S, Schultz A, Civitelli L, Hildesjö C, Larsson M (2018). Alzheimer’ s disease pathology propagation by exosomes containing toxic amyloid - beta oligomers. Acta Neuropathol.

[17] Zheng T, Pu J, Chen Y, Mao Y, Guo Z, Pan H (2017). Plasma exosomes spread and cluster around β -amyloid plaques in an animal model of Alzheimer’ s disease. Front Aging Neurosci.

[18] Fiandaca MS, Kapogiannis D, Mapstone M, Boxer A, Eitan E, Schwartz JB (2015). Identification of preclinical Alzheimer’s disease by a profile of pathogenic proteins in neurally derived blood exosomes: a case-control study. Alzheimers Dement.

[19] Kapogiannis D, Boxer A, Schwartz JB, Abner EL, Biragyn A, Masharani U (2015). Dysfunctionally phosphorylated type 1 insulin receptor substrate in neural-derived blood exosomes of preclinical Alzheimer’s disease. FASEB J.

[20] Mullins RJ, Mustapic M, Goetzl EJ, Kapogiannis D (2017). Exosomal biomarkers of brain insulin resistance associated with regional atrophy in Alzheimer’s disease. Hum Brain Mapp.

[21] Goetzl EJ, Boxer A, Schwartz JB, Abner EL, Petersen RC, Miller BL (2015). Altered lysosomal proteins in neural-derived plasma exosomes in preclinical Alzheimer disease. Neurology.

[22] Goetzl EJ, Kapogiannis D, Schwartz JB, Lobach IV, Goetzl L, Abner EL (2016). Decreased synaptic proteins in neuronal exosomes of frontotemporal dementia and Alzheimer’s disease. FASEB J.

[23] Winston CN, Goetzl EJ, Schwartz JB, Elahi FM, Rissman RA (2019). Complement protein levels in plasma astrocyte-derived exosomes are abnormal in conversion from mild cognitive impairment to Alzheimer’s disease dementia. Alzheimers Dement (Amst)..

[24] Esteban de Antonio E, Pérez-Cordón A, Gil S, Orellana A, Cano A, Alegret M (2021). BIOFACE: a prospective study of risk factors, cognition, and biomarkers in a cohort of individuals with early-onset mild cognitive impairment. study rationale and research protocols. J Alzheimers Dis.

[25] Alegret M, Sotolongo Grau O, De AEE, Cordón AP, Orellana A, Espinosa A (2022). Automatized FACEmemory® scoring is related to Alzheimer’ s disease phenotype and biomarkers in early - onset mild cognitive impairment : the BIOFACE cohort Open Access. Alzheimer’s Res Ther.

[26] Vanderstichele H, Bibl M, Engelborghs S, Le Bastard N, Lewczuk P, Molinuevo JL (2012). Standardization of preanalytical aspects of cerebrospinal fluid biomarker testing for Alzheimer’s disease diagnosis: a consensus paper from the Alzheimer’s biomarkers Standardization Initiative. Alzheimers Dement.

[27] Molinuevo JL, Blennow K, Dubois B, Engelborghs S, Lewczuk P, Perret-Liaudet A (2014). The clinical use of cerebrospinal fluid biomarker testing for Alzheimer’s disease diagnosis: a consensus paper from the Alzheimer’s biomarkers standardization initiative. Alzheimers Dement.

[28] Leitão MJ, Silva-Spínola A, Santana I, Olmedo V, Nadal A, Le BN (2019). Clinical validation of the Lumipulse G cerebrospinal fluid assays for routine diagnosis of Alzheimer’s disease. Alzheimers Res Ther.

[29] Zhang Y, Bi J, Huang J, Tang Y, Du S, Li P (2020). Exosome: a review of its classification, isolation techniques, storage, diagnostic and targeted therapy applications. Int J Nanomed.

[30] Whelan CD, Mattsson N, Nagle MW, Vijayaraghavan S, Hyde C, Janelidze S (2019). Multiplex proteomics identifies novel CSF and plasma biomarkers of early Alzheimer’ s disease. Acta Neuropathol Commun.

[31] Assarsson E, Lundberg M, Holmquist G, Björkesten J, Thorsen SB, Ekman D (2014). Homogenous 96-plex PEA immunoassay exhibiting high sensitivity, specificity, and excellent scalability. PLoS ONE.

[32] Olink©Proteomics. Olink Target 96 Neurology. 2021. https://olink.com/products-services/target/neurology-panel/

[33] Olink©Proteomics. Olink Target 96 Inflammation. 2021. https://olink.com/products-services/target/inflammation/

[34] Orellana A, Garc P, Valero S, Montrreal L, De RI, Hern I (2022). Establishing In-House Cutoffs of CSF Alzheimer’ s Disease Biomarkers for the AT (N) stratification of the Alzheimer Center Barcelona Cohort. Int J Mol Sci.

[35] Sörensen A, Blazhenets G, Schiller F, Meyer PT, Frings L (2020). Amyloid biomarkers as predictors of conversion from mild cognitive impairment to Alzheimer’s dementia: a comparison of methods. Alzheimers Res Ther.

[36] Apostolova LG, Green AE, Babakchanian S, Hwang KS, Chou Y-Y, Toga AW (2012). Hippocampal atrophy and ventricular enlargement in normal aging, mild cognitive impairment (MCI), and Alzheimer Disease. Alzheimer Dis Assoc Disord.

[37] Dubois B, Hampel H, Feldman HH, Scheltens P, Aisen P, Andrieu S (2016). Preclinical Alzheimer’s disease: definition, natural history, and diagnostic criteria. Alzheimers Dement.

[38] DeTure MA, Dickson DW (2019). The neuropathological diagnosis of Alzheimer’s disease. Mol Neurodegener.

[39] Hampel H, Hardy J, Blennow K, Chen C, Perry G, Kim SH (2021). The amyloid-β pathway in Alzheimer’s disease. Mol Psychiatry.

[40] Frontera JA, Boutajangout A, Masurkar AV, Betensky RA, Ge Y, Vedvyas A (2022). Comparison of serum neurodegenerative biomarkers among hospitalized COVID-19 patients versus non-COVID subjects with normal cognition, mild cognitive impairment, or Alzheimer’s dementia. Alzheimers Dement.

[41] Pase MP, Himali JJ, Aparicio HJ, Romero JR, Satizabal CL, Maillard P (2019). Plasma total-tau as a biomarker of stroke risk in the community. Ann Neurol.

[42] Aparicio H, Himali J, Himali D, Romero J, Lioutas V-A, Pase M (2022). Association of plasma nfl levels with risk of cardiovascular disease in the framingham heart study (S33.005). Neurology.

[43] Mustapic M, Eitan E, Werner JK, Berkowitz ST, Lazaropoulos MP, Tran J (2017). Plasma extracellular vesicles enriched for neuronal origin: a potential window into brain pathologic processes. Front Neurosci.

[44] Garcia Cumba LM, Peterson TE, Cepeda MA, Johnson AJ, Parney IF (2019). Isolation and analysis of plasma-derived exosomes in patients with glioma. Front Oncol.

[45] Kumar A, Kim S, Su Y, Sharma M, Kumar P, Singh S (2021). Brain cell-derived exosomes in plasma serve as neurodegeneration biomarkers in male cynomolgus monkeys self-administrating oxycodone. EBioMedicine.

[46] Sharma P, Diergaarde B, Ferrone S, Kirkwood JM, Whiteside TL (2020). Melanoma cell-derived exosomes in plasma of melanoma patients suppress functions of immune effector cells. Sci Rep.

[47] Muller L, Hong C-S, Stolz DB, Watkins SC, Whiteside TL (2014). Isolation of biologically-active exosomes from human plasma. J Immunol Methods.

[48] Okello A, Koivunen J, Edison P, Archer HA, Turkheimer FE, Någren K (2009). Conversion of amyloid positive and negative MCI to AD over 3 years An 11C-PIB PET study. Neurology.

[49] Goetzl EJ, Schwartz JB, Abner EL, Jicha GA, Kapogiannis D (2018). High complement levels in astrocyte-derived exosomes of Alzheimer disease. Ann Neurol.

[50] Goetzl EJ, Abner EL, Jicha GA, Kapogiannis D, Schwartz JB (2018). Declining levels of functionally specialized synaptic proteins in plasma neuronal exosomes with progression of Alzheimer’ s disease. FASEB J.

[51] Ruan Z, Pathak D, Kalavai SV, Yoshii-kitahara A, Bhatt N, Takamatsu-yukawa K (2021). Alzheimer’ s disease brain-derived extracellular vesicles spread tau pathology in interneurons. Brain.

[52] Brinkmalm G, Zetterberg H (2021). The phosphorylation cascade hypothesis of Alzheimer’s disease. Nature Aging.

[53] Forlenza OV, Miranda AS, Guimar I, Talib LL, Diniz BS, Gattaz WF (2015). Decreased neurotrophic support is associated with cognitive decline in non-demented subjects. J Alzheimers Dis.

[54] Straten G, Saur R, Laske C, Gasser T, Annas P, Basun H (2011). Influence of lithium treatment on GDNF serum and CSF concentrations in patients with early Alzheimer’s disease. Curr Alzheimer Res.

[55] Morshed N, Lee MJ, Rodriguez FH, Lauffenburger DA, Mastroeni D, White FM (2021). Quantitative phosphoproteomics uncovers dysregulated kinase networks in Alzheimer’s disease. Nature Aging.

[56] Jonesco DS, Karsdal MA, Henriksen K (2020). The CNS-specific proteoglycan, brevican, and its ADAMTS4-cleaved fragment show differential serological levels in Alzheimer’s disease, other types of dementia and non-demented controls: a cross-sectional study. PLoS ONE.

[57] Minta K, Brinkmalm G, Portelius E, Johansson P, Svensson J, Kettunen P (2021). Brevican and neurocan peptides as potential cerebrospinal fluid biomarkers for differentiation between vascular dementia and Alzheimer’s disease. J Alzheimers Dis.

[58] Tedeschi Dauar M, Picard C, Rosa-Neto P, Villeneuve S, Poirier J (2021). CNTN5 is associated with disease risk and pathology throughout the Alzheimer’s disease continuum. Alzheimers Dement.

[59] Mortamais M, Artero S, Ritchie K (2014). White matter hyperintensities as early and independent predictors of Alzheimer’s disease risk. J Alzheimers Dis.

[60] Moreno-Grau S, de Rojas I, Hernández I, Quintela I, Montrreal L, Alegret M (2019). Genome-wide association analysis of dementia and its clinical endophenotypes reveal novel loci associated with Alzheimer’s disease and three causality networks: the GR@ACE project. Alzheimers Dement.

[61] Nielsen JE, Pedersen KS, Vestergård K, Maltesen RG, Christiansen G, Lundbye-christensen S (2020). Novel blood-derived extracellular vesicle-based proximity extension assay. Biomedicines.

[62] Vom Berg J, Prokop S, Miller KR, Obst J, Kälin RE, Lopategui-Cabezas I (2012). Inhibition of IL-12/IL-23 signaling reduces Alzheimer’s disease-like pathology and cognitive decline. Nat Med.

[63] Pedrini S, Gupta VB, Hone E, Doecke J, O’Bryant S, James I (2017). A blood-based biomarker panel indicates IL-10 and IL-12/23p40 are jointly associated as predictors of β-amyloid load in an AD cohort. Sci Rep.

[64] Johansson P, Almqvist EG, Wallin A, Johansson J-O, Andreasson U, Blennow K (2017). Reduced cerebrospinal fluid concentration of interleukin-12/23 subunit p40 in patients with cognitive impairment. PLoS ONE.

[65] Lin E, Kuo P-H, Liu Y-L, Yang AC, Tsai S-J (2019). Association and interaction effects of interleukin-12 related genes and physical activity on cognitive aging in old adults in the Taiwanese population. Front Neurol.

[66] Nestor SM, Rupsingh R, Borrie M, Smith M, Accomazzi V, Wells JL (2008). Ventricular enlargement as a possible measure of Alzheimer’s disease progression validated using the Alzheimer’s disease neuroimaging initiative database. Brain.

[67] Ertekin T, Acer N, Köseoğlu E, Zararsız G, Sönmez A, Gümüş K (2016). Total intracranial and lateral ventricle volumes measurement in Alzheimer’s disease: a methodological study. J Clin Neurosci.

[68] Walker KA, Hoogeveen RC, Folsom AR, Ballantyne CM, Knopman DS, Windham BG (2017). Midlife systemic inflammatory markers are associated with late-life brain volume. Neurology.

[69] Arevalo-Rodriguez I, Smailagic N, RoquéiFiguls M, Ciapponi A, Sanchez-Perez E, Giannakou A (2015). Mini-Mental State Examination (MMSE) for the detection of Alzheimer’s disease and other dementias in people with mild cognitive impairment (MCI). Cochrane Database Syst Rev.

