# Supplementary material for: Plasma extracellular vesicles reveal early molecular differences in amyloid positive patients with early-onset mild cognitive impairment
Source: J Nanobiotechnology. 2023 Feb 14;21:54. doi: 10.1186/s12951-023-01793-7 (PMC9930227; doi:10.1186/s12951-023-01793-7)
Supplement: Supplementary file 1 — Additional file 1: Figure S1. Neurology panel of Olink Proteomics (code 95801). Distribution of analytical measuring range, defined by the lower and upper limits of quantification (LLOQ-ULOQ), and normal plasma levels where data is available (dark blue bars) for 92 analytes. Figure S2. Inflammation panel of Olink Proteomics (code 95302). Distribution of analytical measuring range, defined by the limits of quantification LLOQ-ULOQ, for 90 out of 92 analytes. Table S1. List of proteins excluded/included in the study after the quality control analysis. Table S2. Demographics and biochemistry of the BIOFACE cohort. Table S3. Pearson correlations and effect between biomarkers in pEVs and most common parameters of AD analysis. Data ordered by R2. Displayed proteins above R2 > 0.3. Table S4. Pearson correlations and effect between biomarkers in CSF and most common parameters of AD analysis. Data ordered by R2. Displayed proteins above R2 > 0.3. Table S5. Pearson correlations and effect between biomarkers in plasma and most common parameters of AD analysis. Data ordered by R2. Displayed proteins above R2 > 0.3. [file 12951_2023_1793_MOESM1_ESM.pdf]

<u>**Supplementary material**</u>

Plasma extracellular vesicles early molecular differences in amyloid positive patients with early-onset mild cognitive impairment

Cano *et al. 2023*

Figures

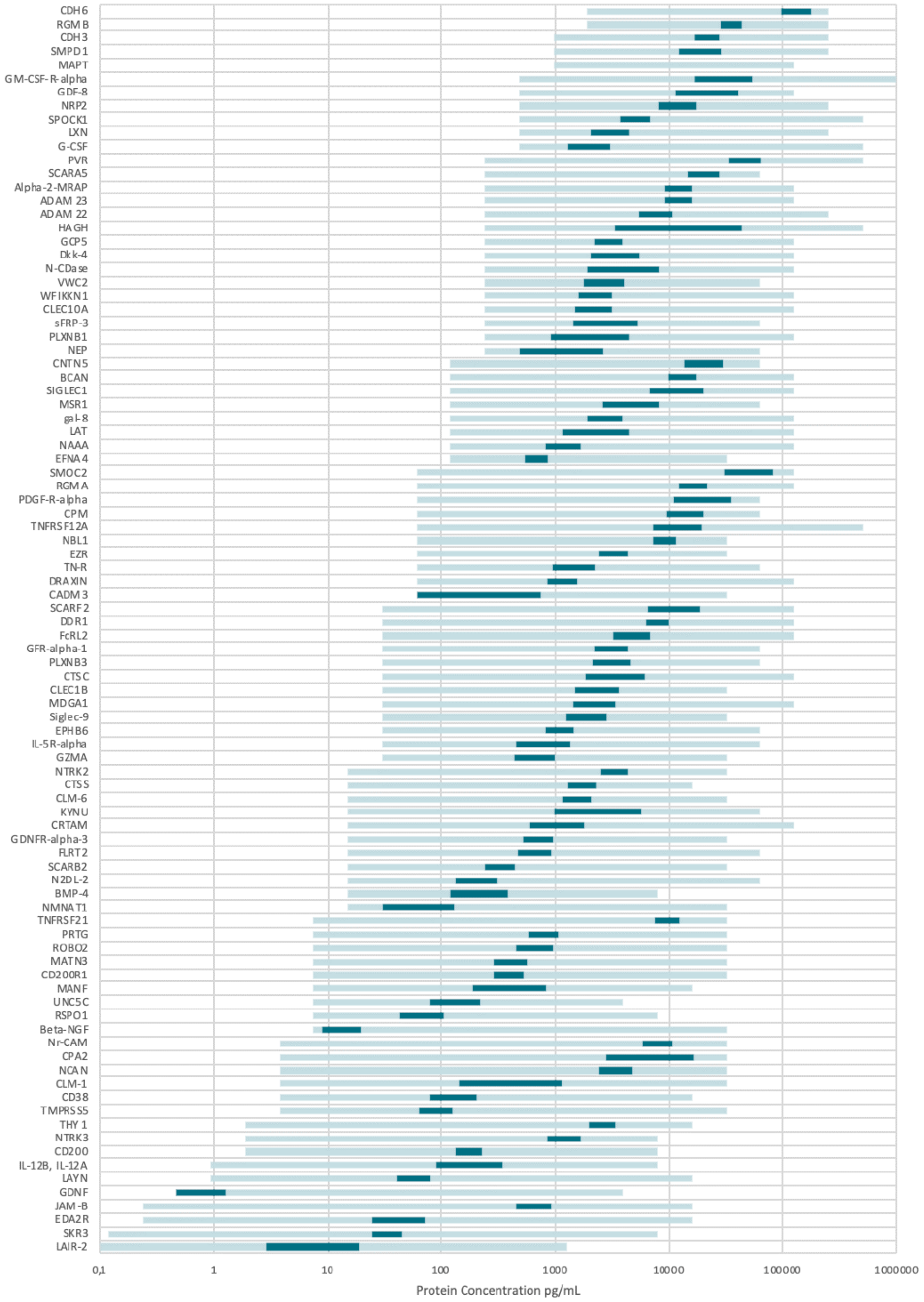

Figure S1. **Neurology panel of Olink Proteomics (code 95801).** Distribution of analytical measuring range, defined by the lower and upper limits of quantification (LLOQ-ULOQ), and normal plasma levels where data is available (dark blue bars) for 92 analytes.

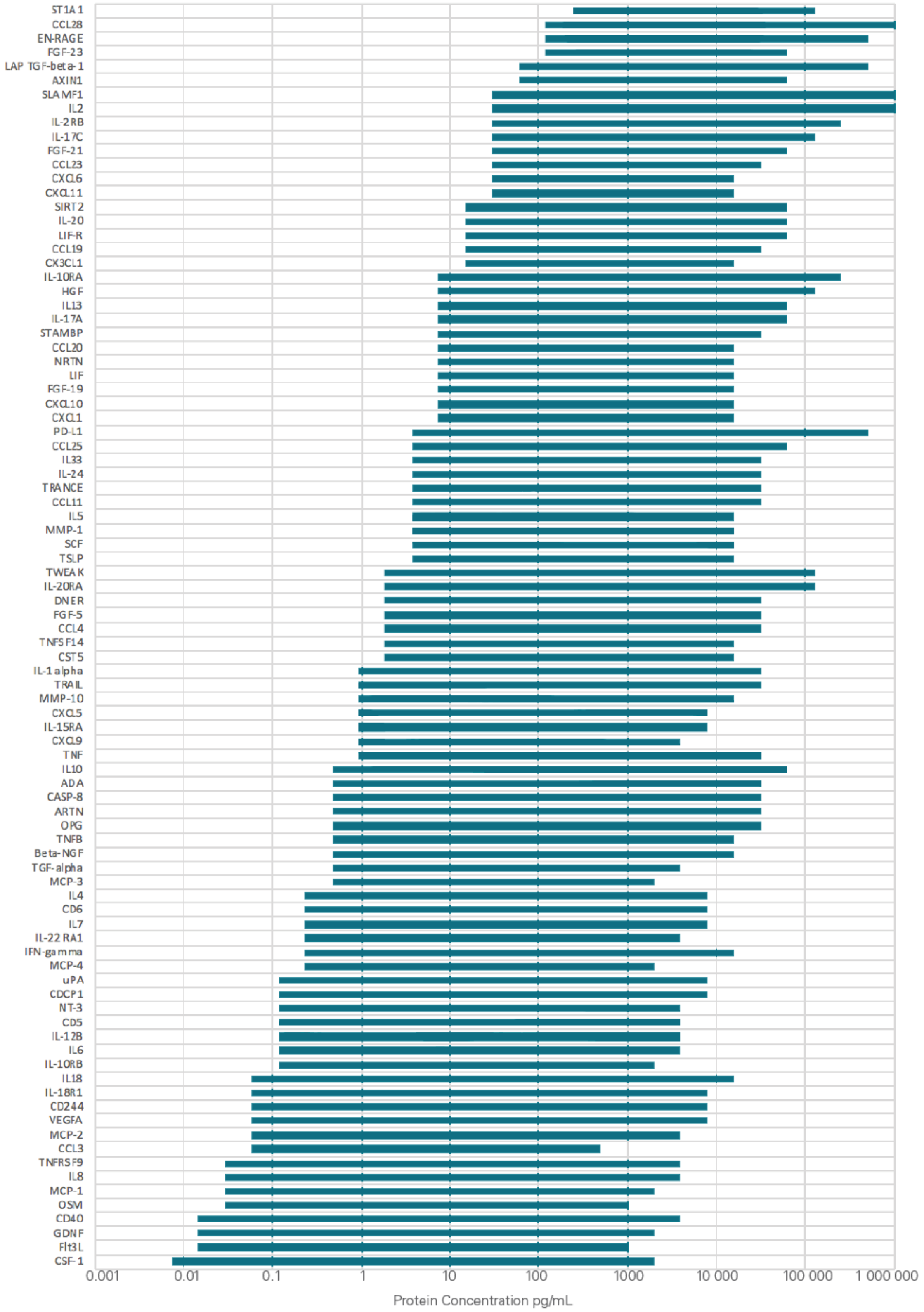

Figure S2. Inflammation panel of Olink Proteomics (code 95302). Distribution of analytical measuring range, defined by the limits of quantification LLOQ-ULOQ, for 90 out of 92 analytes.

Tables

Table S1. List of proteins excluded/included in the study after the quality control analysis.

	Neurology panel						Inflammation panel					
	CSF		Plasma		pEVs		CSF		Plasma		pEVs	
	Excluded	Included	Excluded	Included	Excluded	Included	Excluded	Included	Excluded	Included	Excluded	Included
1	CLEC10A	ADAM.22	MAPT	ADAM.22	ADAM.23	ADAM.22	ARTN	4E.BP1	ARTN	4E.BP1	ARTN	4E.BP1
2	FcRL2	ADAM.23		ADAM.23	Beta.NGF	Alpha.2.MRAP	AXIN1	ADA	Beta.NGF	ADA	Beta.NGF	ADA
3	G.CSF	Alpha.2.MRAP		Alpha.2.MRAP	BMP.4	BCAN	Beta.NGF	CCL11	IL.1.alpha	AXIN1	CASP.8	AXIN1
4	GDNF	BCAN		BCAN	CADM3	CD200	CASP.8	CCL19	IL.20	CASP.8	FGF.23	CCL11
5	LAT	Beta.NGF		Beta.NGF	CDH3	CD200R1	EN.RAGE	CCL20	IL.20RA	CCL11	FGF.5	CCL19
6	LXN	BMP.4		BMP.4	CDH6	CD38	FGF.23	CCL23	IL.22.RA1	CCL19	GDNF	CCL20
7	NMNAT1	CADM3		CADM3	EDA2R	CLEC10A	GDNF	CCL25	IL.24	CCL20	IFN.gamma	CCL23
8		CD200		CD200	EPHB6	CLEC1B	IFN.gamma	CCL28	IL.2RB	CCL23	IL.1.alpha	CCL25
9		CD200R1		CD200R1	FLRT2	CLM.1	IL.1.alpha	CCL3	IL13	CCL25	IL.10RA	CCL28
10		CD38		CD38	G.CSF	CLM.6	IL.10RA	CCL4	IL2	CCL28	IL.15RA	CCL3
11		CDH3		CDH3	MDGA1	CNTN5	IL.15RA	CD244	IL33	CCL3	IL.17A	CCL4
12		CDH6		CDH6	N.CDase	CPA2	IL.17A	CD40	IL4	CCL4	IL.17C	CD244
13		CLEC1B		CLEC10A	NAAA	CPM	IL.17C	CD5	IL5	CD244	IL.20	CD40
14		CLM.1		CLEC1B	TMPRSS5	CRTAM	IL.20	CD6	LIF	CD40	IL.20RA	CD5
15		CLM.6		CLM.1	UNC5C	CTSC	IL.20RA	CD8A	NRTN	CD5	IL.22.RA1	CD6
16		CNTN5		CLM.6		CTSS	IL.22.RA1	CDCP1	TSLP	CD6	IL.24	CD8A
17		CPA2		CNTN5		DDR1	IL.24	CSF.1		CD8A	IL.2RB	CDCP1
18		CPM		CPA2		Dkk.4	IL.2RB	CST5		CDCP1	IL10	CSF.1
19		CRTAM		CPM		DRAXIN	IL10	CX3CL1		CSF.1	IL13	CST5
20		CTSC		CRTAM		EFNA4	IL13	CXCL1		CST5	IL2	CX3CL1
21		CTSS		CTSC		EZR	IL2	CXCL10		CX3CL1	IL33	CXCL1
22		DDR1		CTSS		FcRL2	IL33	CXCL11		CXCL1	IL4	CXCL10
23		Dkk.4		DDR1		gal.8	IL4	CXCL5		CXCL10	IL5	CXCL11
24		DRAXIN		Dkk.4		GCP5	IL5	CXCL6		CXCL11	IL6	CXCL5
25		EDA2R		DRAXIN		GDF.8	MCP.3	CXCL9		CXCL5	IL7	CXCL6
26		EFNA4		EDA2R		GDNF	NRTN	DNER		CXCL6	LIF	CXCL9
27		EPHB6		EFNA4		GDNFR.alpha.3	NT.3	FGF.19		CXCL9	LIF.R	DNER

**28**	EZR	EPHB6	GFR.alpha.1	SLAMF1	FGF.21	DNER	MCP.3	EN.RAGE
**29**	FLRT2	EZR	GM.CSF.R.alpha	ST1A1	FGF.5	EN.RAGE	NRTN	FGF.19
**30**	gal.8	FcRL2	GZMA	TRANCE	Flt3L	FGF.19	SIRT2	FGF.21
**31**	GCP5	FLRT2	HAGH	TSLP	HGF	FGF.21	SLAMF1	Flt3L
**32**	GDF.8	G.CSF	IL.5R.alpha		IL.10RB	FGF.23	TGF.alpha	HGF
**33**	GDNFR.alpha.3	gal.8	IL12		IL.12B	FGF.5	TNF	IL.10RB
**34**	GFR.alpha.1	GCP5	JAM.B		IL.18R1	Flt3L	TRANCE	IL.12B
**35**	GM.CSF.R.alpha	GDF.8	KYNU		IL18	GDNF	TSLP	IL.18R1
**36**	GZMA	GDNF	LAIR.2		IL6	HGF		IL18
**37**	HAGH	GDNFR.alpha.3	LAT		IL7	IFN.gamma		IL8
**38**	IL.5R.alpha	GFR.alpha.1	LAYN		IL8	IL.10RA		LAP.TGF.beta.1
**39**	IL12	GM.CSF.R.alpha	LXN		LAP.TGF.beta.1	IL.10RB		MCP.1
**40**	JAM.B	GZMA	MANF		LIF	IL.12B		MCP.2
**41**	KYNU	HAGH	MAPT		LIF.R	IL.15RA		MCP.4
**42**	LAIR.2	IL.5R.alpha	MATN3		MCP.1	IL.17A		MMP.1
**43**	LAYN	IL12	MSR1		MCP.2	IL.17C		MMP.10
**44**	MANF	JAM.B	N2DL.2		MCP.4	IL.18R1		NT.3
**45**	MAPT	KYNU	NBL1		MMP.1	IL10		OPG
**46**	MATN3	LAIR.2	NCAN		MMP.10	IL18		OSM
**47**	MDGA1	LAT	NEP		OPG	IL6		PD.L1
**48**	MSR1	LAYN	NMNAT1		OSM	IL7		SCF
**49**	N.CDase	LXN	Nr.CAM		PD.L1	IL8		ST1A1
**50**	N2DL.2	MANF	NRP2		SCF	LAP.TGF.beta.1		STAMBP
**51**	NAAA	MATN3	NTRK2		SIRT2	LIF.R		TNFB
**52**	NBL1	MDGA1	NTRK3		STAMBP	MCP.1		TNFRSF9
**53**	NCAN	MSR1	PDGF.R.alpha		TGF.alpha	MCP.2		TNFSF14
**54**	NEP	N.CDase	PLXNB1		TNF	MCP.3		TRAIL
**55**	Nr.CAM	N2DL.2	PLXNB3		TNFB	MCP.4		TWEAK
**56**	NRP2	NAAA	PRTG		TNFRSF9	MMP.1		uPA
**57**	NTRK2	NBL1	PVR		TNFSF14	MMP.10		VEGFA
**58**	NTRK3	NCAN	RGMA		TRAIL	NT.3		
**59**	PDGF.R.alpha	NEP	RGMB		TWEAK	OPG		
**60**	PLXNB1	NMNAT1	ROBO2		uPA	OSM		

**61**	PLXNB3	Nr.CAM	RSPO1	VEGFA	PD.L1
**62**	PRTG	NRP2	SCARA5		SCF
**63**	PVR	NTRK2	SCARB2		SIRT2
**64**	RGMA	NTRK3	SCARF2		SLAMF1
**65**	RGMB	PDGF.R.alpha	sFRP.3		ST1A1
**66**	ROBO2	PLXNB1	Siglec.9		STAMBP
**67**	RSPO1	PLXNB3	SIGLEC1		TGF.alpha
**68**	SCARA5	PRTG	SKR3		TNF
**69**	SCARB2	PVR	SMOC2		TNFB
**70**	SCARF2	RGMA	SMPD1		TNFRSF9
**71**	sFRP.3	RGMB	SPOCK1		TNFSF14
**72**	Siglec.9	ROBO2	THY.1		TRAIL
**73**	SIGLEC1	RSPO1	TN.R		TRANCE
**74**	SKR3	SCARA5	TNFRSF12A		TWEAK
**75**	SMOC2	SCARB2	TNFRSF21		uPA
**76**	SMPD1	SCARF2	VWC2		VEGFA
**77**	SPOCK1	sFRP.3	WFIKKN1		
**78**	THY.1	Siglec.9			
**79**	TMPRSS5	SIGLEC1			
**80**	TN.R	SKR3			
**81**	TNFRSF12A	SMOC2			
**82**	TNFRSF21	SMPD1			
**83**	UNC5C	SPOCK1			
**84**	VWC2	THY.1			
**85**	WFIKKN1	TMPRSS5			
**86**		TN.R			
**87**		TNFRSF12A			
**88**		TNFRSF21			
**89**		UNC5C			
**90**		VWC2			
**91**		WFIKKN1			

Table S2. Demographics and biochemistry of the BIOFACE cohort.

Parameters	EOMCI Aβ42(+)		EOMCI Aβ42(-)		Welch t' test			
	mean	SD(±)	mean	SD(±)	Unpaired t test with Welch's correction		F test to compare variances	
n	18		62		p Value	p Summary	p Value	p Summary
Female (%)	61		66					
Male (%)	39		34					
Age LP	61,551	3,459	61,059	3,574				
MMSE	27,588	2,181	27,918	1,828				
Aβ42_CSF (pg/ml)	611,222	131,206	1159,258	319,344	<0,0001	****	0,000	***
p-Tau181_CSF (pg/ml)	67,278	59,340	46,952	20,891	0,170	ns	<0,0001	****
t-Tau_CSF (pg/ml)	415,444	365,773	293,806	178,269	0,188	ns	<0,0001	****
Qalb (pg/ml)	0,884	0,092	0,890	0,102	0,789	ns	0,627	ns
Glucose_CSF (mg/dL)	64,111	8,983	63,919	12,409	0,943	ns	0,139	ns
Glucose_Blood (mg/dL)	105,833	20,092	106,822	19,598	0,855	ns	0,841	ns
Total protein_CSF (g/L)	0,449	0,106	0,450	0,163	0,965	ns	0,050	*
Total protein_Serum (g/L)	68,400	4,209	69,806	3,695	0,212	ns	0,450	ns
Total Protein_pEVs (μg/ml)	1581,142	578,180	1335,728	377,106	0,040	*	0,001	***
Albumin_CSF (%)	55,011	5,950	54,431	5,703	0,716	ns	0,770	ns
Total globulins_CSF (%)	40,122	6,376	40,471	5,888	0,837	ns	0,627	ns
Albumin:globulins index_CSF	1,437	0,449	1,396	0,362	0,722	ns	0,221	ns
Alpha 1 globulins_CSF (%)	3,417	1,021	4,277	1,609	0,009	**	0,040	*
Alpha 2 globulins_CSF (%)	7,428	1,872	8,152	2,299	0,181	ns	0,350	ns
Beta globulins_CSF (%)	17,533	3,979	17,726	3,849	0,857	ns	0,807	ns
Albumin_serum (%)	62,339	3,503	61,294	3,625	0,278	ns	0,920	ns
Total globulins_serum (%)	37,661	3,503	38,706	3,625	0,278	ns	0,920	ns
Albumin:globulins index_serum	1,677	0,234	1,605	0,242	0,266	ns	0,928	ns
Alpha 1 globulins_serum (%)	3,772	0,542	3,681	0,459	0,520	ns	0,340	ns
Alpha 2 globulins_serum (%)	10,133	1,505	9,735	1,370	0,323	ns	0,575	ns
Beta globulins_serum (%)	10,906	1,095	11,506	1,163	0,053	ns	0,816	ns
Gamma globulins_serum (%)	12,850	2,552	13,784	2,882	0,1945	ns	0,5928	ns
Hemoglobin (g/dL)	13,938	1,170	13,501	1,331	0,186	ns	0,571	ns

**Leucocites (/µL)**	6026,111	1837,243	5966,935	1426,006	0,901	ns	0,153	ns
**Eosinophils (/µL)**	182,062	83,351	185,852	88,921	0,868	ns	0,800	ns
**Basophils (/µL)**	35,644	15,634	35,085	16,617	0,896	ns	0,814	ns
**Linfocites (/µL)**	1708,337	514,209	1939,127	542,775	0,109	ns	0,841	ns
**Monocites (/µL)**	424,767	123,528	418,159	124,476	0,843	ns	-	-
**Neutrophils (/µL)**	3675,301	1480,189	3388,712	1069,071	0,452	ns	0,067	ns
**Mean corpuscular hemoglobin (pg)**	30,597	1,824	30,187	1,727	0,4027	ns	0,7206	ns
**Medium corpuscular volume (fL)**	92,179	4,454	91,660	4,483	0,667	ns	-	-
**Hematocrit (%)**	42,000	3,373	41,002	3,917	0,2952	ns	0,5034	ns
**Red Blood Cell Dispersion Index**	14,456	1,430	14,666	1,630	0,5984	ns	0,5629	ns
**Red Blood Cells count (/µL)**	4565555,556	435825,413	4477258,065	409029,917	0,4499	ns	0,6878	ns
**Platelet count (/µL)**	235944,444	63504,027	225758,065	56665,740	0,5451	ns	0,5038	ns

Table S3. Pearson correlations and effect between biomarkers in pEVs and most common parameters of AD analysis. Data ordered by R^2. Displayed proteins above $R^2 > 0.3$.

**pEVs Aβ42(+)**					
**Neurology**			**Inflammation**		
	**Correlation (Pearson r)**	**Effect (R^2)**		**Correlation (Pearson r)**	**Effect (R^2)**
**Ptau**			**Ptau**	-	-
**GDNFR.alpha.3**	-0.6620	0.4382			
**BCAN**	-0.6196	0.3840			
**DDR1**	-0.6179	0.3818			
**SMPD1**	-0.6178	0.3817			
**PDGF.R.alpha**	-0.5947	0.3536			
**CNTN5**	-0.5761	0.3319			
**SPOCK1**	-0.5532	0.3060			
**Aβ42**	-	-	**Aβ42**	-	-
**Age**	-	-	**Age**		
			**CD40**	0.5741	0.3296
**MMSE**	-	-	**MMSE**		
			**TNFB**	-0.6037	0.3644
			**CDCP1**	-0.5979	0.3575
**Qalb**			**Qalb**	-	-
**ADAM.22**	0.5670	0.3215			
**Sex**			**Sex**		
			**TNFRSF9**	-0.5489	0.3013

**pEVs Aβ42(-)**					
**Neurology**			**Inflammation**		
	**Correlation (Pearson r)**	**Effect (R^2)**		**Correlation (Pearson r)**	**Effect (R^2)**
**Ptau**	-	-	**Ptau**	-	-
**Aβ42**	-	-	**Aβ42**	-	-
**Age**	-	-	**Age**	-	-
**MMSE**	-	-	**MMSE**	-	-
**Qalb**	-	-	**Qalb**	-	-
**Sex**	-	-	**Sex**	-	-

Table S4. Pearson correlations and effect between biomarkers in CSF and most common parameters of AD analysis. Data ordered by R^2. Displayed proteins above $R^2 > 0.3$.

**CSF Aβ42(+)**					
**Neurology**			**Inflammation**		
	**Correlation (Pearson r)**	**Effect (R^2)**		**Correlation (Pearson r)**	**Effect (R^2)**
**Ptau**			**Ptau**		
**MAPT**	0.9603	0.9222	**VEGFA**	0.8757	0.7669
**CNTN5**	0.8463	0.7163	**SCF**	0.8743	0.7644
**EPHB6**	0.8456	0.7150	**CSF.1**	0.858	0.7362
**Beta.NGF**	0.8252	0.6809	**LIF.R**	0.8348	0.6969
**EDA2R**	0.8117	0.6589	**uPA**	0.8291	0.6874
**RGMB**	0.8004	0.6406	**TWEAK**	0.8291	0.6873
**Alpha.2.MRAP**	0.7816	0.6109	**FGF.5**	0.8183	0.6695
**NTRK2**	0.7715	0.5951	**ADA**	0.8072	0.6516
**NTRK3**	0.7583	0.5751	**4E.BP1**	0.7885	0.6217
**CLM.6**	0.7208	0.5196	**HGF**	0.785	0.6163
**UNC5C**	0.7200	0.5184	**CD40**	0.7729	0.5973

**ROBO2**	0.7166	0.5135	**IL.10RB**	0.7623	0.5812
**Dkk.4**	0.7069	0.4997	**PD.L1**	0.7475	0.5588
**MANF**	0.7022	0.4931	**STAMBP**	0.7352	0.5405
**Siglec.9**	0.702	0.4927	**TGF.alpha**	0.7331	0.5374
**ADAM.23**	0.701	0.4914	**TNFRSF9**	0.733	0.5373
**PVR**	0.7006	0.4908	**SIRT2**	0.731	0.5343
**SCARB2**	0.6962	0.4847	**Flt3L**	0.7199	0.5182
**gal.8**	0.6951	0.4831	**OPG**	0.6739	0.4542
**PLXNB1**	0.6883	0.4737	**FGF.19**	0.6696	0.4483
**PLXNB3**	0.6849	0.4690	**TNFB**	0.6653	0.4427
**CDH6**	0.6673	0.4453	**IL8**	0.6603	0.4361
**PRTG**	0.6670	0.4449	**CD8A**	0.6578	0.4328
**TN.R**	0.6570	0.4316	**CX3CL1**	0.6533	0.4268
**SMOC2**	0.6546	0.4286	**TRAIL**	0.6365	0.4052
**GCP5**	0.6536	0.4272	**MMP.10**	0.6214	0.3862
**EZR**	0.6419	0.412	**CCL23**	0.6188	0.3829
**LAYN**	0.6392	0.4085	**CCL19**	0.6024	0.3629
**SPOCK1**	0.6242	0.3896	**CXCL5**	0.5774	0.3334
**MATN3**	0.6217	0.3865	**CDCP1**	0.5767	0.3326
**MDGA1**	0.6201	0.3845	**DNER**	0.5751	0.3307
**JAM.B**	0.6114	0.3738	**IL18**	0.5521	0.3048
**GFR.alpha.1**	0.6095	0.3715			
**GDF.8**	0.6063	0.3676			
**SKR3**	0.6054	0.3665			
**TNFRSF12A**	0.5612	0.3149			
**Aβ42**	-	-	**Aβ42**		
			**CD244**	-0.9255	0.8566
			**CD6**	-0.7088	0.5024
**Age**			**Age**		
**LAIR.2**	0.6321	0.3995	**OPG**	0.6295	0.3963
**CLM.6**	0.5479	0.3002	**TNFB**	0.6071	0.3686
			**CD40**	0.6017	0.3620
			**ADA**	0.6013	0.3616
			**TWEAK**	0.5975	0.3571
			**MCP.1**	0.568	0.3227
			**MCP.2**	0.5616	0.3154
			**HGF**	0.5608	0.3145
			**Flt3L**	0.5583	0.3117
			**uPA**	0.5513	0.3039
**MMSE**	-	-	**MMSE**		
			**MCP.4**	0.6001	0.3601
**Qalb**	-	-	**Qalb**		
			**OSM**	-0.8131	0.6611
			**CD244**	0.6007	0.3608
			**FGF.19**	-0.5728	0.3281
			**IL7**	-0.5642	0.3183
			**CD40**	-0.5472	0.2994
**Sex**			**Sex**		
**RSPO1**	-0.7009	0.4912	**CD244**	-0.718	0.5155
**HAGH**	-0.6497	0.4220			
**WFIKKN1**	-0.5927	0.3513			
**IL.5R.alpha**	-0.5482	0.3005			

CSF Aβ42(-)	
**Neurology**	**Inflammation**

	Correlation (Pearson r)	Effect (R^2)		Correlation (Pearson r)	Effect (R^2)
**Ptau**			**Ptau**		
**MAPT**	0.9103	0.8287	**SCF**	0.8338	0.6953
**Beta.NGF**	0.8659	0.7497	**VEGFA**	0.7137	0.5094
**EPHB6**	0.8372	0.7008	**CSF.1**	0.6688	0.4474
**CNTN5**	0.813	0.6609	**LIF.R**	0.6199	0.3843
**CDH6**	0.8075	0.652	**SIRT2**	0.6162	0.3796
**SCARB2**	0.8022	0.6436	**ADA**	0.6079	0.3696
**NTRK2**	0.8014	0.6422	**IL.10RB**	0.5609	0.3146
**ADAM.23**	0.7993	0.6389	**FGF.5**	0.551	0.3036
**LAYN**	0.7755	0.6014			
**RGMB**	0.748	0.5595			
**PVR**	0.7421	0.5507			
**Alpha.2.MRAP**	0.728	0.5300			
**GFR.alpha.1**	0.7178	0.5152			
**Dkk.4**	0.6985	0.4879			
**GCP5**	0.6708	0.4499			
**NTRK3**	0.6575	0.4323			
**HAGH**	0.6371	0.4059			
**SMOC2**	0.5941	0.353			
**ROBO2**	0.5804	0.3369			
**CLM.6**	0.58	0.3364			
**UNC5C**	0.5757	0.3314			
**PDGF.R.alpha**	0.5551	0.3081			
**EDA2R**	0.5504	0.3029			
**EFNA4**	0.5483	0.3006			
**Aβ42**			**Aβ42**	-	-
**SCARB2**	0.6355	0.4039			
**LAYN**	0.603	0.3636			
**CDH6**	0.6002	0.3602			
**PDGF.R.alpha**	0.5867	0.3442			
**GCP5**	0.5617	0.3155			
**ADAM.23**	0.5592	0.3127			
**NTRK2**	0.5468	0.2990			
**Age**	-	-	**Age**	-	-
**MMSE**	-	-	**MMSE**	-	-
**Qalb**	-	-	**Qalb**	-	-
**Sex**	-	-	**Sex**	-	-

Table S5. Pearson correlations and effect between biomarkers in plasma and most common parameters of AD analysis. Data ordered by R^2. Displayed proteins above $R^2 > 0.3$.

**Plasma Aβ42(+)**					
**Neurology**			**Inflammation**		
	**Correlation (Pearson r)**	**Effect (R^2)**		**Correlation (Pearson r)**	**Effect (R^2)**
**Ptau**			**Ptau**		
Beta.NGF	0.6526	0.4258	IL.10RA	0.7403	0.5481
			CCL11	-0.6712	0.4505
			IL.17C	-0.5919	0.3504
			CCL20	-0.5722	0.3274
			FGF.23	0.5720	0.3272
**Aβ42**			**Aβ42**	-	-
TN.R	-0.6029	0.3635			
SPOCK1	-0.5713	0.3264			

Neurology	Correlation (Pearson r)	Effect (R^2)	Inflammation	Correlation (Pearson r)	Effect (R^2)
**Age**			**Age**		
LXN	-0.8488	0.7204	CCL19	-0.7915	0.6264
HAGH	0.7910	0.6257	CDCP1	0.5837	0.3408
GZMA	-0.5524	0.3052	IL.10RA	-0.5804	0.3368
**MMSE**	-	-	**MMSE**		
			OSM	-0.6638	0.4406
			TNFSF14	-0.6435	0.4141
**Qalb**	-	-	**Qalb**		
			SLAMF1	0.6594	0.4348
			FGF.5	-0.6546	0.4284
			IL.10RB	0.6286	0.3952
**Sex**	-	-	**Sex**	-	-

**Plasma Aβ42(-)**					
**Neurology**			**Inflammation**		
	**Correlation (Pearson r)**	**Effect (R^2)**		**Correlation (Pearson r)**	**Effect (R^2)**
**Ptau**	-	-	**Ptau**	-	-
**Aβ42**	-	-	**Aβ42**	-	-
**Age**	-	-	**Age**	-	-
**MMSE**	-	-	**MMSE**	-	-
**Qalb**	-	-	**Qalb**	-	-
**Sex**	-	-	**Sex**	-	-
